# Low Dose BCG Infection as a Model for Macrophage Activation Maintaining Cell Viability

**DOI:** 10.1155/2016/4048235

**Published:** 2016-10-19

**Authors:** Leslie Chávez-Galán, Dominique Vesin, Denis Martinvalet, Irene Garcia

**Affiliations:** ^1^Department of Pathology and Immunology, Centre Medical Universitaire (CMU), Faculty of Medicine, University of Geneva, Geneva, Switzerland; ^2^Laboratory of Integrative Immunology, National Institute of Respiratory Diseases “Ismael Cosio Villegas”, Mexico City, Mexico; ^3^Department of Cell Physiology and Metabolism, Centre Medical Universitaire (CMU), Faculty of Medicine, University of Geneva, Geneva, Switzerland

## Abstract

*Mycobacterium bovis* BCG, the current vaccine against tuberculosis, is ingested by macrophages promoting the development of effector functions including cell death and microbicidal mechanisms. Despite accumulating reports on* M. tuberculosis*, mechanisms of BCG/macrophage interaction remain relatively undefined. In vivo, few bacilli are sufficient to establish a mycobacterial infection; however, in vitro studies systematically use high mycobacterium doses. In this study, we analyze macrophage/BCG interactions and microenvironment upon infection with low BCG doses and propose an in vitro model to study cell activation without affecting viability. We show that RAW macrophages infected with BCG at MOI 1 activated higher and sustained levels of proinflammatory cytokines and transcription factors while MOI 0.1 was more efficient for early stimulation of IL-1*β*, MCP-1, and KC. Both BCG infection doses induced iNOS and NO in a dose-dependent manner and maintained nuclear and mitochondrial structures. Microenvironment generated by MOI 1 induced macrophage proliferation but not MOI 0.1 infection. In conclusion, BCG infection at low dose is an efficient in vitro model to study macrophage/BCG interactions that maintains macrophage viability and mitochondrial structures. This represents a novel model that can be applied to BCG research fields including mycobacterial infections, cancer immunotherapy, and prevention of autoimmunity and allergies.

## 1. Introduction 

*Mycobacterium tuberculosis* (*M. tuberculosis*), the causative agent of tuberculosis (TB) infection, is a major global health problem including transmission of drug-resistant strains (MDR-TB) and the increased risk of TB among HIV-infected persons [1]. The World Health Organization estimated that in 2013 there were 9 million cases of TB and 1.5 million died from the disease. TB cases coinfected with HIV were around 1.1 million with an estimated 480,000 new cases of MDR-TB. This report indicates the need to intensify the efforts in TB control and to give access to high-quality care for all TB patients [2].

*Mycobacterium bovis Bacillus Calmette-Guérin* (BCG), the current vaccine used against TB, is a live attenuated mycobacterium which was isolated in 1908 and administrated for the first time to newborn infants in 1921 [3]. BCG genome contains a deletion in the region of differentiation 1 (RD1) and its cytolytic activity is decreased compared to* M. tuberculosis *[4, 5]. The BCG strains used in clinics were derived from the original BCG, for example, BCG Pasteur, Danish, Japan, Moreau, Tice, and Connaught; each vaccine has its own immunogenic ability in both animals and humans [6, 7]. BCG vaccine provides effective protection against childhood TB but the level of protection against adult pulmonary TB can be variable [8]. To date, there are significant advances in development of new vaccines against TB; however so far, BCG remains the only licensed vaccine to prevent TB and over 2 billion people have been immunized with BCG.

Currently, BCG clinical application is not limited to the field of mycobacterial infections. Adjuvant instillation of BCG as standard immunotherapy used in non-muscle-invasive bladder cancer (NMIBC) results in a significant reduction in tumor and progression. It has been described that high doses of BCG Connaught induce a high Th1 response, prime CD8+ T cells, and efficiently prevent recurrences of NMIBC [9]. However, the optimal dose and duration of treatment in NMIBC are controversial and derived side effects may lead to premature interruption of treatment. New treatment schedules of BCG with no negative impact on patients are considered [10].

Macrophages are part of mononuclear phagocytic system, and the alveolar macrophage is the first cell to encounter* M. tuberculosis* after infection; however the bacillus has the ability to evade microbicidal activities. For example,* M. tuberculosis *inhibits phagolysosomal fusion; consequently, macrophage provides a niche for mycobacteria and although it is a hostile environment, it is sufficient for bacillus growth [11, 12].

Using human monocytes and macrophages and macrophage cell lines, it has been shown that avirulent strains of mycobacteria induce higher levels of apoptosis than virulent strains which represent a mechanism of host defense which can be subverted by virulent* M. tuberculosis* to escape from innate immunity [13–15]. However, data have also reported that virulent and avirulent* M. tuberculosis* strains can both inhibit and promote apoptosis according to experimental conditions [16–19]. Despite accumulating data on macrophage activation and death by* M. tuberculosis *strains, BCG-mediated cellular activities in phagocytic cells remain relatively unexplored. Studies comparing BCG with* M. tuberculosis* have frequently used high Multiplicity-of-Infection (MOI). In general, studies use BCG infection at MOI 10 (10 bacilli/cell) pointing apoptosis as a cellular strategy to eliminate mycobacteria [20, 21].

BCG-induced activation of macrophages has been associated with TNF production which is a critical cytokine in host defense mechanisms against mycobacterial infection [22]. Both BCG-infected and uninfected macrophages produce TNF although in lower amount than macrophages infected with virulent strains in which TNF supports mycobacterial growth [23]. BCG-induced TNF activates iNOS but TNF can control intracellular BCG growth by iNOS-dependent and iNOS-independent pathways [24]. Collectively, these models have provided important insights into immune response against mycobacteria but considering that a low dose of bacteria (1 bacillus) is sufficient to establish a mycobacterial infection in the host, it is of interest to analyze macrophage responses in terms of cell death, activation, and integrity to low number of mycobacteria [25].

In the present work, we compare RAW macrophage responses upon infection with BCG Pasteur at MOI 1 and MOI 0.1; the last is considered as a very low dose of infection. This study aims (1) to evaluate if low doses of BCG Pasteur are sufficient to activate macrophages while maintaining their viability and mitochondrial integrity and (2) to determine if under low MOIs the macrophage is able to retain their functional activities over time after infection. Our results show that low BCG doses are an efficient* in vitro* infectious model to study interactions between macrophages/BCG maintaining viable macrophages and mitochondrial integrity.

## 2. Material and Methods

### 2.1. Cell Culture

The murine macrophage cell line RAW 264.7 (RAW macrophages) was purchased from American Type Culture Collection (Rockville, MD). The cells were maintained in DMEM supplemented with 10% head-inactivated FBS, penicillin, streptomycin, sodium pyruvate, glutamine, and HEPES (complete DMEM) at 37°C in a humidified atmosphere containing 5% CO_2_.

### 2.2. *M. bovis* BCG

*M. bovis* BCG Pasteur strain 1172 P2 (Pasteur Institute, Paris, France) was used and grown to the log phase in 7H9 middlebrook medium supplemented with oleic albumin dextrose catalase (OADC). The bacteria were then harvested, washed, and frozen at −80°C in PBS plus 10% of glycerol. Bacterial load was determined by plating serial 10-fold dilutions on 7H10 middlebrook agar (supplemented with OADC) and counting colonies after incubation for at least 3 weeks [26]. A BCG-GFP* M. bovis* BCG Pasteur strain harboring phsp60-gfp expressing Green Fluorescence Protein (BCG-GFP) was grown in the presence of Kanamycin as previously reported [27].

### 2.3. BCG Infection

RAW macrophages were cultured in 24-well flat-bottomed cell culture plates (1 × 10^6^/mL) and infected with BCG Pasteur at MOI 1 or MOI 0.1 along 2, 5, 18, 24, 30, 48, and 72 hours (h) at 37°C in a humidified atmosphere containing 5% CO_2_.

### 2.4. Evaluation of Cell Death by Flow Cytometry

Ending the culture, cells were harvested by adding cold PBS to culture plates and maintaining for 10 minutes on ice to detach cells from plastic plates. Cells were washed in PBA (phosphate buffered saline containing 0.1% Sodium Azide and 0.1% Albumin Bovine) and incubated with 3 *μ*L 7-AAD (eBioscience) solution for 20 min at 4°C in the dark, washed in PBA, suspended in binding buffer 1x (BD pharmingen), and incubated with 5 *μ*L Annexin-V FITC-conjugated or APC-conjugated (when using BCG-GFP infection) (eBioscience) for 15 min at room temperature in the dark. Data were collected using a FACs CyAn (Beckton Dickinson, Inc.) within one hour and then analyzed with FlowJo software (Tree Star, Inc.). 50,000 events were acquired per sample.

### 2.5. Flow Cytometry

The percentage of RAW macrophages expressing transmembrane TNF (tmTNF) and intracellular TNF (iTNF) was assessed by flow cytometry. Briefly, for iTNF, ending the culture, cells were harvested, washed in PBA, and fixed in 4% formaldehyde for 10 min at 4°C. Cells were incubated with saponin-containing buffer, shaken gently for 10 min at 4°C, and then incubated with anti-TNF (MP6-XT22) conjugated PE Cy7 (eBioscience) for 30 min at room temperature in the dark and washed with PBS-saponin. For tmTNF label, cells were harvested, washed in PBA, and fixed in 4% formaldehyde; RAW macrophages were suspended in PBA and stained with anti-TNF 30 min at 4°C and washed with PBA. Fluorochrome-labeled isotype-matched control antibody was used to evaluate background staining. After incubation with antibodies, cells were washed twice in PBA and data collected using a FACs CyAn and analyzed with FlowJo software. 100,000 events were acquired per sample.

### 2.6. Cytokine and Chemokine Measurements

At different time points after BCG infection, cell-free supernatants were collected and frozen at −80°C for cytokine or chemokine assessment. Cytokine amounts were assessed by ELISA for TNFR1, TNFR2, TNF, IL-6, IL-1*β*, MIP-1*α*, MCP-1, and KC, in accordance with the manufacturer's instructions.

### 2.7. Nitrite Assay

Cell-free supernatants were collected at different time points after BCG infection and nitrite content was measured using Griess method as previously described [28]. The plate was incubated with Griess reagent (sulfonamide 1% plus N-(1-naphthyl)-ethylenediamine dihydrochloride 0.1% in phosphoric acid 2.5%) at room temperature for 5 min in the dark. Absorbance at 570 nm was measured with a microplate reader. NO concentrations were calculated using a standard curve.

### 2.8. Western Blotting

Ending the culture, cells were harvested, washed in PBS, and lysed in RIPA buffer containing protease inhibitor (Complete Mini Protease Inhibitor Cocktail Tablet, Roche). Cellular protein extracts were separated by SDS-PAGE and transferred to 0.2 *μ*m pore-size nitrocellulose membranes (Bio-Rad Laboratories, Hercules, CA, USA) with 25 mm Tris-base (pH 8.0) containing 150 mm glycine and 20% (volume/volume) methanol as previously described [28]. Membranes were incubated with antibodies to phospho-p44/42 MAPK (ERK1/2), phospho-nuclear factor kappa-light-chain-enhancer of activated B cells (NF*κ*B p65), phospho-apoptosis signal-regulating kinase 1 (ASK1) (Ser967) (from Cell Signaling Technology), rabbit polyclonal anti-iNOS (Calbiochem Merck), and anti-caspase-1 (clone 5B10) (BioLegend). Protein bands were detected by incubating with horseradish peroxidase-labeled antibodies and visualized with enhanced chemiluminescence reagent (Advantas) using an ImageQuant Las-4000 mini (GE Healthcare Life Science). Band densities were analyzed by densitometry using the online IMAGEJ 1.39c software (National Institutes of Health) (http://rsb.info.nih.gov/ij/index.html) as described by Luke Miller (http://www.lukemiller.org/journal/2007/08/quantifying-western-blots-without.html). Samples were normalized using tubulin as loading control.

### 2.9. Macrophage Proliferation Assay

RAW macrophage proliferation was determined using the Click-iT® EdU Flow Cytometry assay kit (Invitrogen™ Life Technology Inc.) in accordance with the manufacturer's instructions. Briefly, fresh RAW macrophages were cultured in 24-well flat-bottomed cell culture plates (1 × 10^6^/mL) in DMEM/F-12 medium supplemented (volume/volume) with supernatants recovered from different time points and MOI cultures of infected macrophages. Three *μ*M of EdU (5-ethylnyl-2′-deoxyuridine) was added to cultures and cells were left for 12 or 24 h in culture, at 37°C in a humidified atmosphere containing 5% CO_2_. Cells were harvested, fixed, permeabilized, and stained with Alexa Fluor 488 dye for detention of DNA synthesis. As control of proliferation, RAW macrophages cultured in DMEM/F-12 medium was used. Data were collected using a FACs CyAn, analyzed with FlowJo software. 50,000 events were acquired per sample.

### 2.10. Transfection of RAW Cells and Analysis by Confocal Microscopy

RAW macrophages were transfected with plasmid encoding mitochondrial targeted red fluorescein protein (mitoRFP) at final concentration of 3 *μ*g plasmid/1 × 10^6^ cells to generate RAW-mitoRFP. We used a Neon Transfection System under conditions, 1680 v (pulse voltage), 20 ms (pulse width), and 1 (pulse number), and maintained in DMEM culture medium plus G418 antibiotic (500 *μ*g/mL) (Mediatech, Inc., Manassas, VA). Transfected macrophages were sorted based on the expression of mitoRFP using a FACs-Aria II; we obtained 97% of RAW cells which were RAW-mitoRFP^+^. Sorted RAW-mitoRFP^+^ cells were then infected with BCG-GFP at MOI 0.1 and MOI 1. Cultures were done on Chamber Slide System (Thermo Scientific, Inc., Waltham, MA) and maintained 2, 5, or 18 h at 37° in a humidified atmosphere containing 5% CO_2_. Cells were washed with PBS and fixed in 4% formaldehyde during 15 minutes and mounted with mounting medium containing DAPI (Vector Laboratories, Inc., Burlingame, CA). The slides were examined by SP5 confocal microscopy, and the Leica Application Suite (LAS) software was used for analysis (Leica Microsystems, Co).

### 2.11. Statistical Analysis

Results are expressed as means ± SEM. Data comparisons were performed using one-way ANOVA followed by a Dunnett's* post hoc* test for multiple comparisons. Two-tailed unpaired Student's *t*-test was used to evaluate differences between 2 independent groups (GraphPad Software, Inc., San Diego, CA). A *P* value <0.05 was considered to be statistically significant.

## 3. Results

### 3.1. BCG Infection at MOI 1 but Not MOI 0.1 Induced Cell Death

We have first assessed early and late apoptosis and necrosis in RAW macrophages induced by BCG Pasteur infection using MOI 1 (1 bacillus/cell) versus MOI 0.1 (1 bacillus/10 cells) by flow cytometry. Studies comparing BCG with* M. tuberculosis* have frequently used high MOI. In general, studies have used BCG infection at MOI 10 (10 bacilli/cell) showing that apoptosis is a cell strategy to eliminate mycobacteria [20, 21].

**Figure 1:**
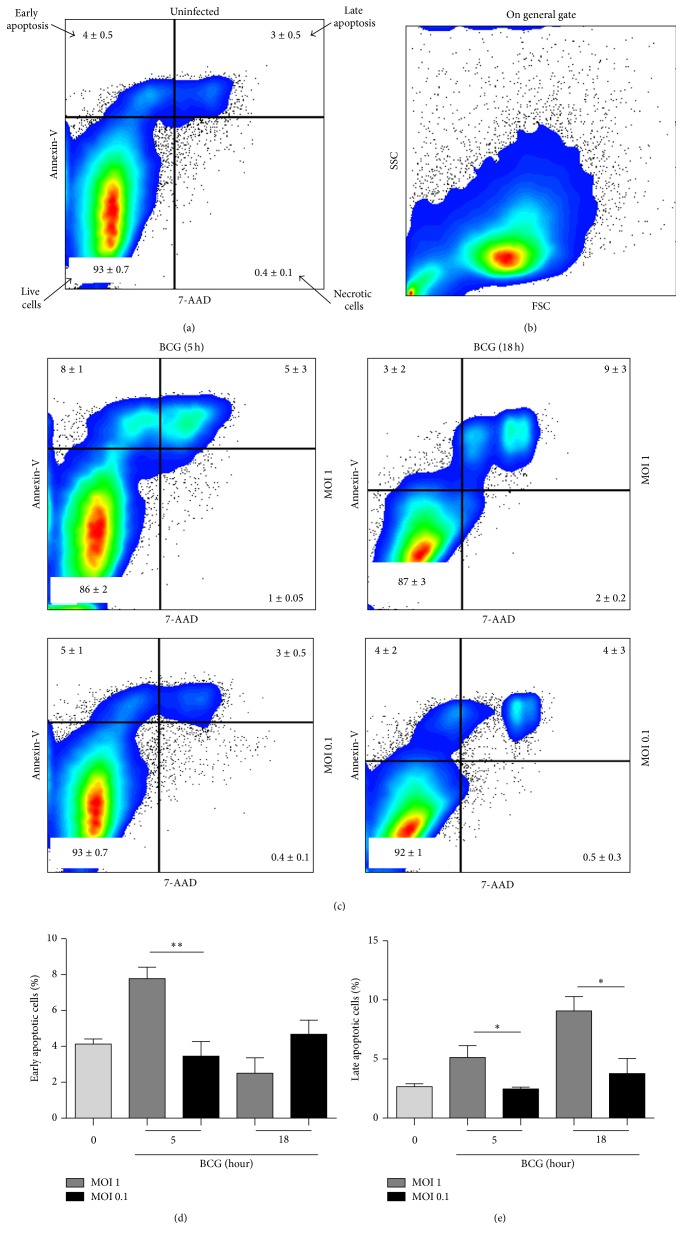
BCG infection at MOI 1 but not MOI 0.1 induces cell death. (a) Uninfected RAW macrophages were cultured or, (b) and (c), infected with BCG Pasteur at MOI 1 and MOI 0.1 during 2, 5, and 18 hours and stained with Annexin-V and 7-AAD to assess the percentage of cell death by flow cytometry as shown in the representative plots. (b) Representative FCS and SSC scatter plots of cells infected for 5 h with MOI 1 and (c) Annexin-V versus 7-AAD at 5 and 18 h. Percentage of early (d) and late (e) apoptosis induced by BCG at 5 and 18 h. Two-tailed unpaired Student's *t*-test was used to compare percentage of infected cells MOI 1 versus MOI 0.1. Bars indicate mean ± SEM from four independent experiments. ^*∗∗*^ *P* < 0.01; ^*∗*^ *P* < 0.05. Light grey refers to uninfected cells.

Cells were analyzed in a dot plot color where Annexin-V^+^ cells indicated early apoptotic cells, Annexin-V^+^7-AAD^+^ late apoptotic cells, 7-AAD^+^ necrotic cells, and negative cells for both markers were living cells as using a general gate (Figures 1(a) and 1(b)). At MOI 1, we observed an increase of early apoptosis at 5 h after infection whereas late apoptosis increased at 5 h and 18 h, contrasting with MOI 0.1 infected cells which did not exhibit apoptosis after infection compared to uninfected cells (Figures 1(c), 1(d), and 1(e)).

### 3.2. BCG Infection at MOI 1 and MOI 0.1 Activated the Expression of tmTNF, sTNF, and sTNF Receptors

TNF is a major proinflammatory cytokine induced by BCG infection and can be observed intracellularly (iTNF), on the cell surface as transmembrane (tmTNF) and in a soluble TNF form (sTNF) [22, 29–32].* In vivo*, BCG infection has shown that tmTNF may interact with soluble TNF receptors (sTNFR1 or sTNFR2) which may play a critical role in the infection outcome [32].

**Figure 2:**
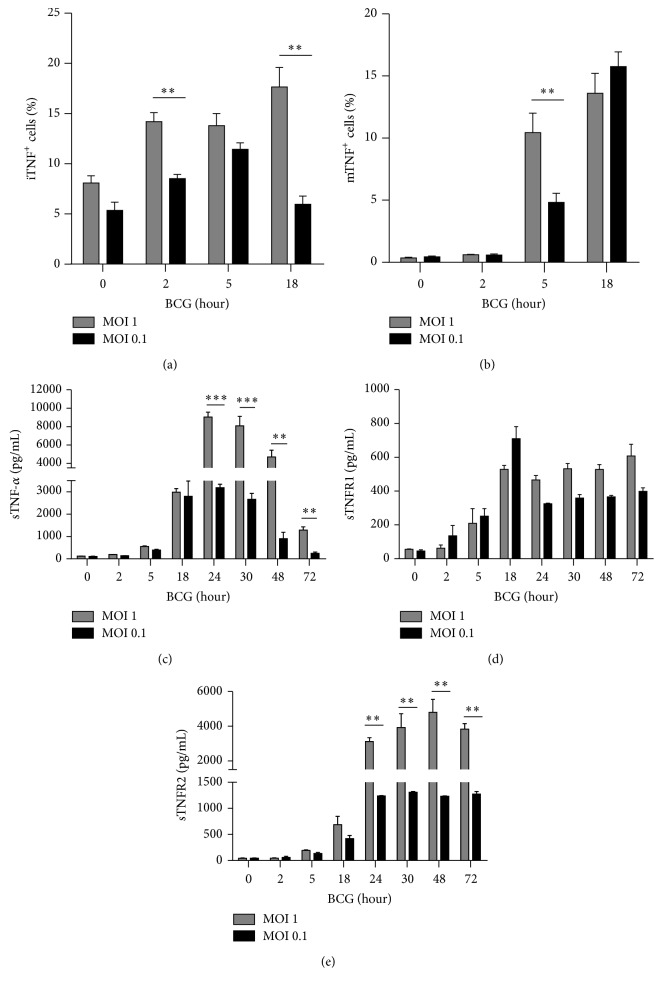
BCG infection at MOI 1 and MOI 0.1 activates TNF and soluble TNF receptors. (a) and (b) RAW macrophages were infected at MOI 1 or MOI 0.1 during 2, 5, and 18 h and stained for intracellular and transmembrane TNF with mAb against TNF. (c) Concentrations of soluble form of TNF were assessed by ELISA. (d) and (e) Soluble TNFR1 and TNFR2 were measured by ELISA in culture supernatant from RAW macrophages infected with BCG Pasteur at MOI 1 and MOI 0.1. Two-tailed unpaired Student's *t*-test was used to evaluate statistical differences. Bars indicate mean ± SEM from four independent experiments in each case. ^*∗∗∗*^ *P* < 0.001; ^*∗∗*^ *P* < 0.01; ^*∗*^ *P* < 0.05.

To determine if low doses of BCG were enough to activate TNF and sTNFRs, we measured iTNF and tmTNF by flow cytometry and sTNF, sTNFR1, and sTNFR2 levels by ELISA. Our data showed that both MOI 1 and MOI 0.1 activated iTNF and responses were BCG dose dependent. The main difference between MOIs was that iTNF expression was more transient using MOI 0.1 compared to MOI 1 (Figure 2(a)). However, the percentage of MOI 1 infected cells expressing tmTNF was higher than cells infected with MOI 0.1 at 5 hrs but similar at 18 hrs after infection (Figure 2(b)). We then assessed sTNF and observed induction in a dose dependent manner and the levels were maintained longer with MOI 1 than with MOI 0.1 (Figure 2(c)). Regarding sTNFRs, we observed that sTNFR1 was similarly regulated by the two MOIs, but in contrast sTNFR2 expression was BCG dose dependent (Figures 2(d) and 2(e)). Collectively, our data show that both MOI 1 and MOI 0.1 induce iTNF, sTNF, and sTNFR2 in a dose dependent manner but expression levels of tmTNF and sTNFR1 appear independent of MOIs.

### 3.3. Different Activation Patterns of Cytokines and Chemokines Triggered by BCG Infection at MOI 1 and MOI 0.1

**Figure 3:**
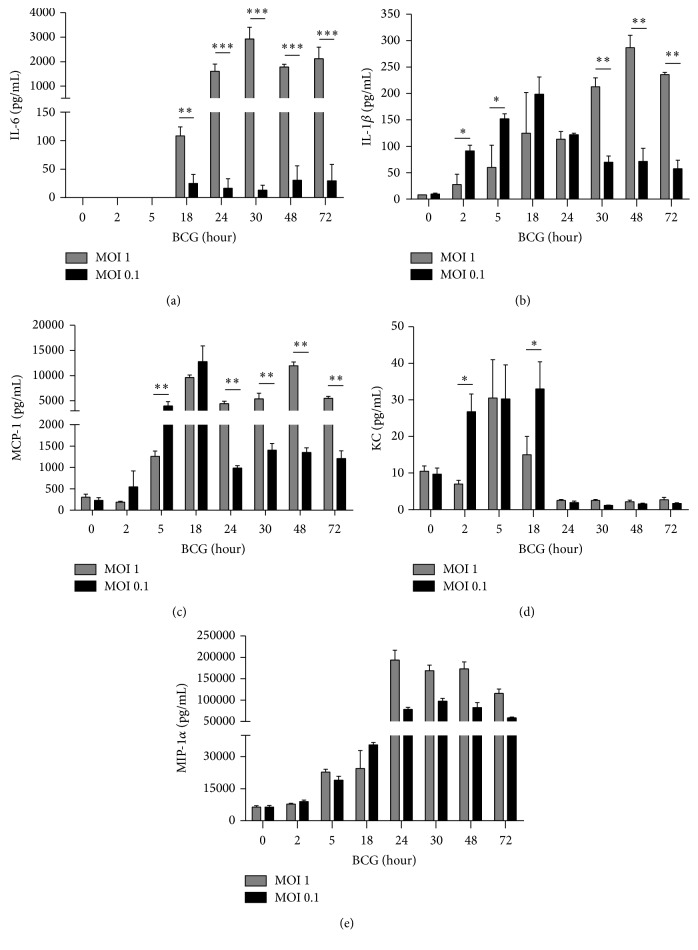
BCG infection at MOI 1 and MOI 0.1 is efficient to induce cellular activation but kinetics are different. (a) Concentrations of proinflammatory cytokines IL-6, (b) IL-1*β*, and chemokines, (c) MCP-1, (d) KC, and (e) MIP-1*α*, were measured by ELISA in culture supernatant from infected RAW macrophages with BCG Pasteur at MOI 1 and MOI 0.1 during different time points. Bars indicate mean ± SEM from four independent experiments. ANOVA and Dunnett's* post hoc* test compared to uninfected macrophages or MOI 1 versus MOI 0.1. ^*∗∗∗*^ *P* < 0.001, ^*∗∗*^ *P* < 0.01, and ^*∗*^ *P* < 0.05.

Secretion of cytokines other than TNF as well as chemokines is necessary for cellular activation and recruitment following mycobacterial infection* in vivo*. We have then compared the levels of secreted cytokines and chemokines after BCG infection and observed that IL-6 is only activated with MOI 1 but not with MOI 0.1 (Figure 3(a)). Unexpectedly, IL-1*β* was produced earlier with MOI 0.1 than with MOI 1 and peaked at 18 h after infection and decreased at 24 h after infection. In contrast, MOI 1 induced IL-1*β* which reached a maximum at 48 h but this high level was maintained up to 72 h (Figure 3(b)). Western blot analyses showed an increased level at 2 h after infection of procaspase-1 in cells infected with MOI 0.1 compared with those infected with MOI 1. However, amounts of caspase-1 p20 subunit were not significantly different using MOI 1 and MOI 0.1 (see Supplementary Figure 1 in Supplementary Material available online at http://dx.doi.org/10.1155/2016/4048235). We have assessed the concentration of three chemokines, monocyte chemotactic protein 1 (MCP-1), chemokine (C-X-C motif) ligand 1 or also called keratinocyte chemoattractant (KC), and macrophage inflammatory protein-1 alpha (MIP-1*α*) which have important chemoattractant activity for monocytes and neutrophils [33]. BCG infection at MOI 0.1 induced high levels of MCP-1 from 5 h and still increased at 18 h but at 24 h MCP-1 was found downregulated. Conversely, MOI 1 induced high level at 18 h that was maintained until 72 h after infection (Figure 3(c)). KC was induced at 2 h by MOI 0.1 and in higher levels than MOI 1 infection (Figure 3(d)). Both MOI 1 and MOI 0.1 increased MIP-1*α* level up to 5 h after infection (Figure 3(e)). Our data show that MOI 0.1 is efficient to induce early IL-1*β* and caspase-1 activation and that cytokine and chemokine kinetics induced by MOI 1 versus MOI 0.1 is different suggesting that intracellular pathways are regulated by infection dosages.

### 3.4. Differential Regulation of Phosphorylated ASK1 and NF*κ*B by BCG Infection at MOI 1 and MOI 0.1

**Figure 4:**
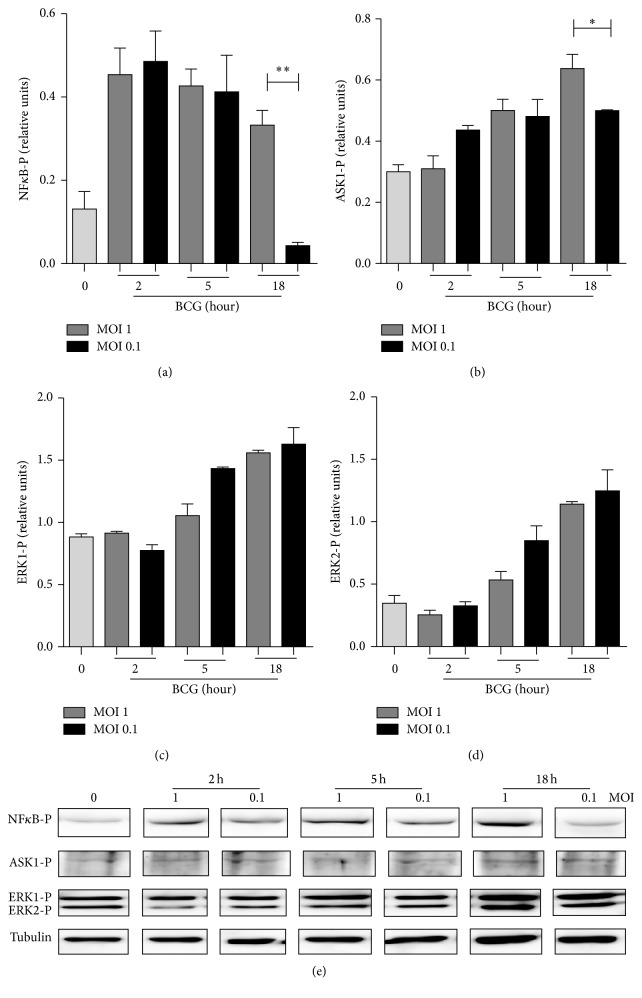
BCG infection at MOI 1 and MOI 0.1 activates NF*κ*B and MAPK pathways. (a) Phosphorylated forms of NF*κ*B, (b) ASK, (c) ERK1, and (d) ERK2 were evaluated by western blot analyses. Band densities were normalized against tubulin by densitometry analyses. (e) Representative western blot from three independent experiments. Results are shown in relative units of concentration using IMAGEJ software. Two-tailed unpaired Student's *t*-test was used to evaluate statistical differences. Bars indicate mean ± SEM. ^*∗∗*^ *P* < 0.01; ^*∗*^ *P* < 0.05. Light grey bar indicates uninfected cells (0).

Following our previous results indicating that MOI 1 and MOI 0.1 may activate different intracellular pathways, we examined transcription factors involved in cytokine expression including nuclear factor kappa B (NF*κ*B) phosphorylation required for cytokine transcription [34]. We assessed extracellular signal-regulated kinase (ERK1/2) and apoptosis signal-regulating kinase (ASK1), two members of the MAPK family involved in inflammatory process [35, 36]. Levels of phosphorylated NF*κ*B (NF*κ*B-P), ASK1 (ASK1-P), ERK1 (ERK1-P), and ERK2 (ERK2-P) were determined by western blot analyses at different time points after BCG infection. We observed that MOI 1 and MOI 0.1 similarly induced phosphorylation of NF*κ*B-P and ASK1-P and signaling was sustained with MOI 1 but not with MOI 0.1 (Figures 4(a) and 4(b)). However, ERK1-P and ERK2-P pathways showed the same pattern of activation which was independent of BCG infection doses (Figures 4(c) and 4(d)). Together, these data show that phosphorylated NF*κ*B and ASK correlate with soluble cytokine expression while ERK1-P and ERK2-P are activated similarly with both low and high BCG doses.

### 3.5. Microenvironment Produced by BCG Infection at MOI 1 Induces Effects on Macrophage Proliferation

**Figure 5:**
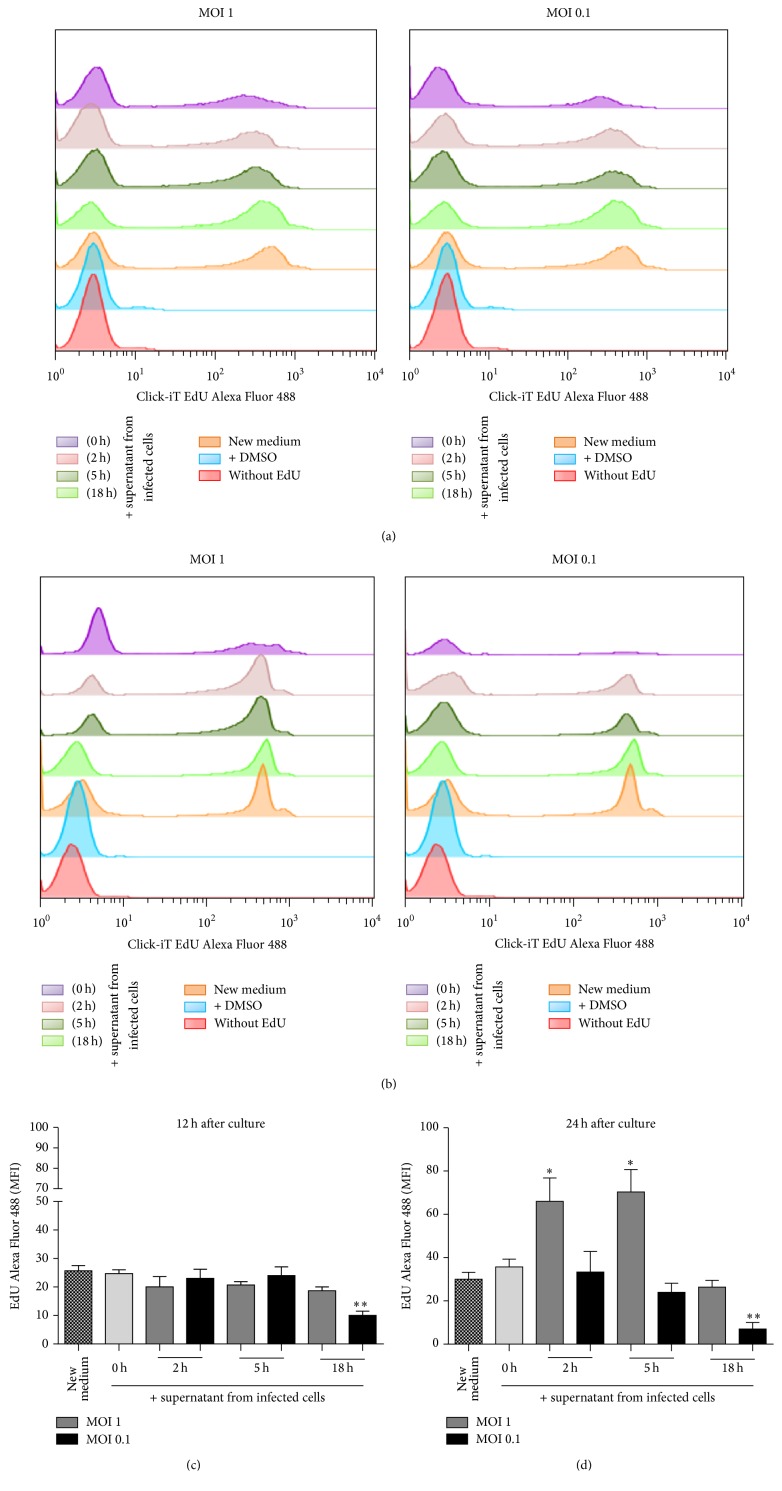
Microenvironment produced by BCG infection at MOI 1 affects macrophage proliferation. (a) and (b) Representative histograms measuring mean fluorescence intensity (MFI) of Alexa Fluor 488 after 12 h and 24 h. (c) and (d) Supernatant from infected cells was recovered at different time points after infection and added to fresh RAW macrophages and proliferation was evaluated at 12 h and 24 h in the presence of EdU. RAW macrophages were stained with Alexa Fluor 488 and proliferation was evaluated by flow cytometry. Two-tailed unpaired Student's *t*-test was used to evaluate differences versus macrophages proliferation in new medium. Bars indicate mean ± SEM from three independent experiments. ^*∗∗*^ *P* < 0.01; ^*∗*^ *P* < 0.05. The light grey bar refers to uninfected cells or 0 h.

Our data showing that cellular activation is MOI- and time-dependent could suggest that the microenvironment generated by the infection would affect macrophage proliferation. To test this hypothesis, we recovered the supernatant from infected cells at different time points which was added to fresh RAW macrophages and cell proliferation was evaluated after 12 h and 24 h as previously described [37]. Macrophages cultured in fresh medium were considered as control cells for proliferation. Macrophage proliferation at 12 h was not affected by supernatant of BCG-infected cells at MOI 1 but was inhibited by supernatant of 18 h of BCG-infected cells at MOI 0.1 (Figures 5(a) and 5(b)). In contrast, macrophage proliferation at 24 hours was significantly increased with 2 and 5 h supernatant of BCG-infected cells at MOI 1, while again supernatant of 18 h of BCG-infected cells at MOI 0.1 inhibited the macrophage proliferation (Figures 5(c) and 5(d)). Thus, these results show that microenvironment generated with MOI 1 enhanced cell proliferation but not MOI 0.1.

### 3.6. Low Dose BCG Infection Induced Microbicidal Effector Functions

**Figure 6:**
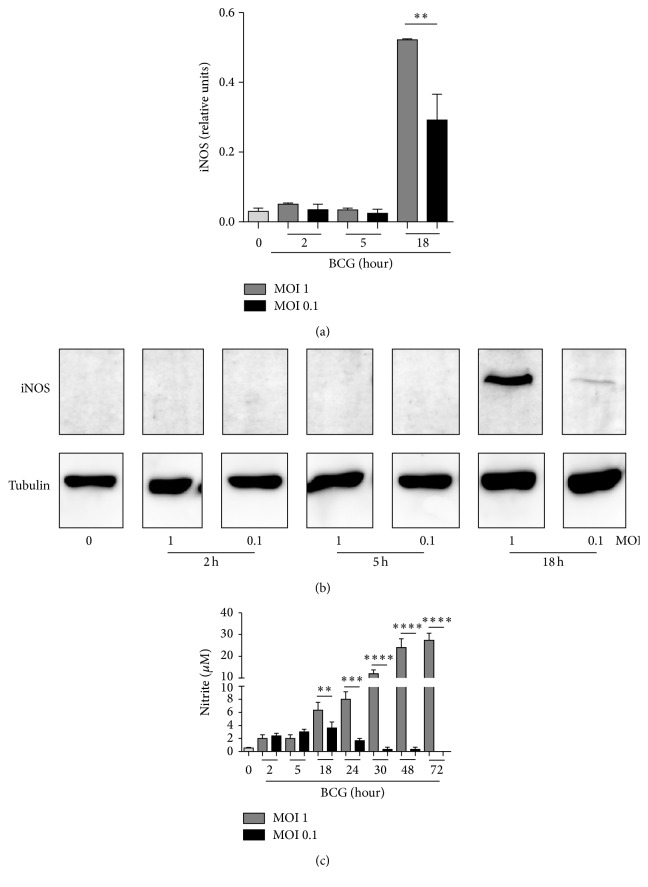
BCG infection at MOI 1 and MOI 0.1 induces iNOS and NO production but only MOI 1 maintains this function for long time. RAW macrophages were infected with BCG Pasteur at MOI 1 and MOI 0.1. (a) Cells were recovered at 2, 5, and 18 h after infection and iNOS expression was evaluated by western blot. Band densities were normalized against tubulin by densitometry analysis. Results are shown in relative units of concentration using IMAGEJ software. (b) Representative western blot for iNOS and tubulin as a loading control. (c) Nitrite was evaluated in culture supernatant from each condition using Griess reagent. Two-tailed unpaired Student's *t*-test was used to evaluate differences versus uninfected cells. Bars indicate mean ± SEM from three (a) or five (c) independent experiments. ^*∗∗∗∗*^ *P* < 0.0001, ^*∗∗∗*^ *P* < 0.001, and ^*∗∗*^ *P* < 0.01. Grey bar refers to uninfected cells indicated by 0.

Activation of inducible nitric oxide synthase (iNOS) to generate oxide nitric (NO) is an important effector mechanism to eliminate mycobacteria [38]. We compared macrophage iNOS expression by western blot in cells infected with BCG at MOI 1 and MOI 0.1. Infection at MOI 1 induced higher levels of iNOS protein than MOI 0.1 at 18 h after infection (Figures 6(a) and 6(b)). We also measured the levels of nitrite as an indirect manner to evaluate NO production. We observed that although NO was similarly produced at early infection, infection with BCG at MOI 1 yielded sustained and higher NO levels at later time points. Infection with BCG at MOI 0.1 triggered lower and transient NO production (Figure 6(c)). Our data showed that both MOIs activate NO production; however only MOI 1 maintains longer and higher NO levels at late time points.

### 3.7. BCG Infection with Low Bacterial Burden Does Not Alter Mitochondrial Distribution and Nuclear Integrity

**Figure 7:**
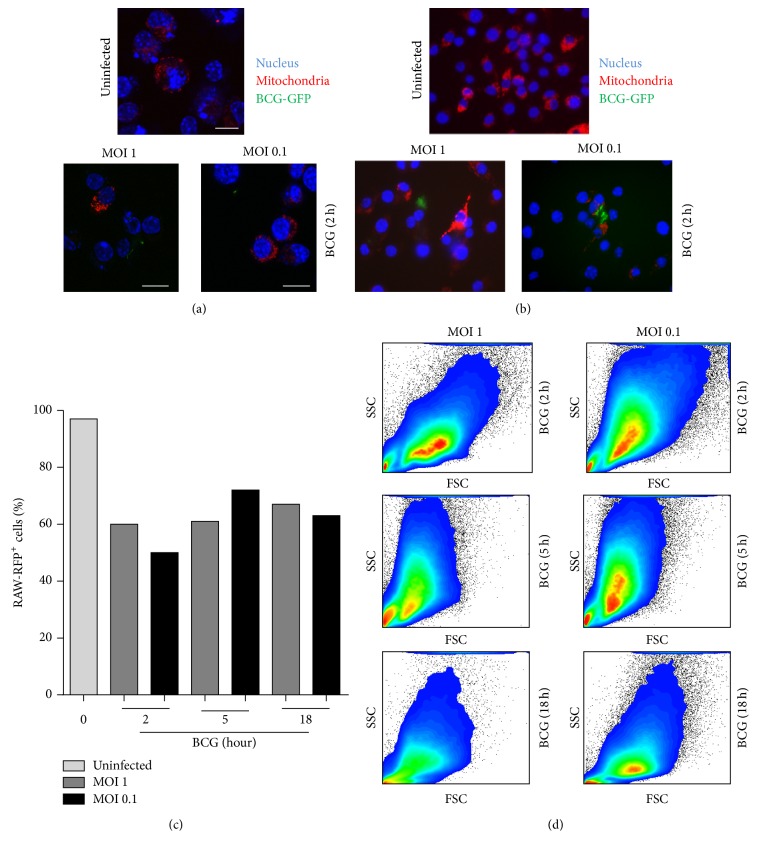
BCG infection at MOI 0.1 and MOI 1 maintains undisturbed mitochondrial and nuclear integrity. (a) Transfected RAW-mitoRFP macrophages and (b) transfected and sorted (enriched to 97% or RAW-mitoRFP^+^) were infected at MOI 1 and MOI 0.1 BCG-GFP. Slides were prepared with DAPI and mounting medium and analyzed by confocal SP5 microscopy using Leica Application Suite software. Mitochondria (red), BCG (green), and nucleus (blue) (left panel). Scale bar 10 *μ*m. (c) RAW-mitoRFP cells were sorted, enriched to 97% (RAW-mitoRFP^+^), and infected with BCG-GFP and quantified for RAW-mitoRFP^+^ cells before and after BCG-GFP infection at MOI 1 and MOI 0.1. (d) Flow cytometry analyses of cell integrity and size using side scatter (SSC)/forward scatter (FSC). Representative image from three independent experiments.

**Figure 8:**
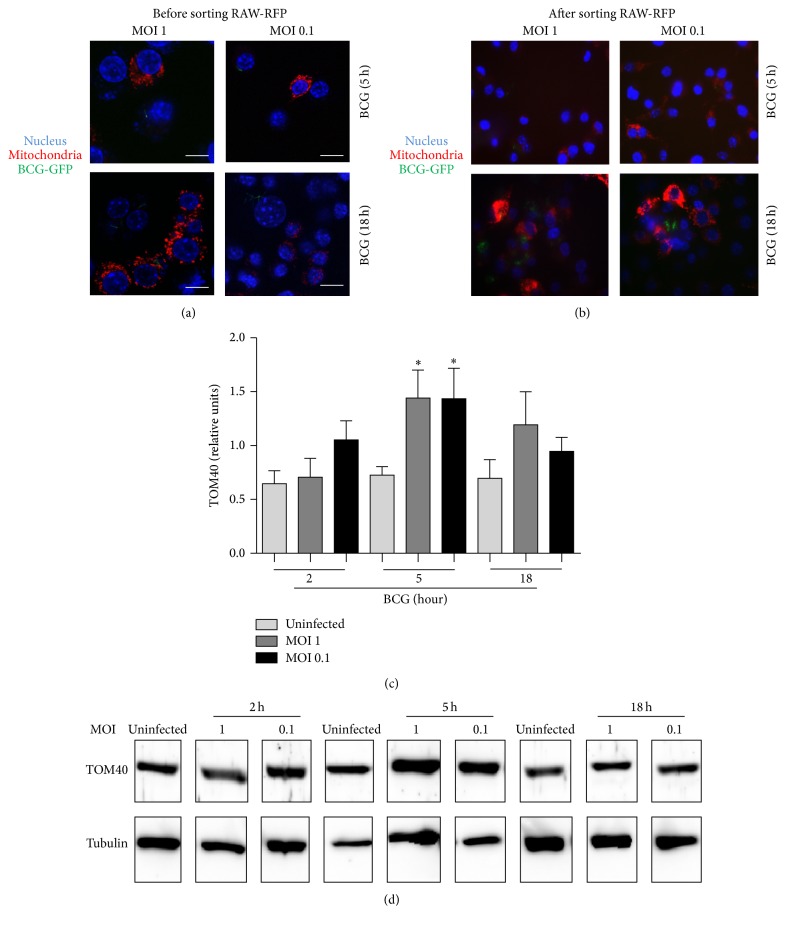
Amount of TOM40 is maintained at both MOI 1 and MOI 0.1. (a) RAW-mitoRFP macrophages were infected with BCG-GFP at MOI 1 and MOI 0.1 for 5 and 18 h or (b) sorted for mitoRFP and then infected. (c) Western blots for TOM40, band densities were normalized against tubulin. (d) Representative western blot from three independent experiments. Two-tailed unpaired Student's *t*-test was used to evaluate statistical differences. Bars indicate mean ± SEM from three experiments. ^*∗*^ *P* < 0.05.

Host cell death mediated by infectious agents involves modulation of mitochondria. Using* M. tuberculosis *infection model, it has been reported that high dose (MOI ≥ 10) induced cell death where mitochondria and nucleus were the first organelles showing damage [39, 40]. We asked if BCG infection with low dose affects mitochondrial integrity. To explore this question, RAW macrophages were transfected with mitoRFP (RAW-mitoRFP), infected with BCG-GFP, and examined by confocal microscopy to evaluate nuclear structure and localization of mitochondria. Results showed that mitochondrial distribution and nuclear integrity after BCG-GFP infection were maintained in a similar form than in uninfected cells (Figures 7(a) and 7(b)). We observed that the number of RAW-mitoRFP cells decreased after the infection. To better examine this result, RAW-mitoRFP^+^ cells were sorted and enriched to 97% and then infected with BCG-GFP (Figures 8(a) and 8(b)). A reduction of 25–40% of mitoRFP^+^ cells was still observed (Figure 7(c)). In addition, we observed that cell integrity and size assessed by flow cytometry were similar in infected and uninfected cells, but only a discrete modification of the cell structure was observed at 18 h MOI 1 postinfection (Figure 7(d)). In order to evaluate if the decrease of mitoRFP expression was due to mitochondrial loss, we measured the presence of the constitutive mitochondrial molecule TOM40 by western blot analyses. TOM40 expression only increased at 5 h after infection with BCG at both MOIs indicating that loss of mitoRFP expression was not due to loss of mitochondrial mass during BCG infection (Figures 8(c) and 8(d)). Together, these results show that low doses of BCG do not affect the nuclear integrity, mitochondrial quantity, or distribution, even if MOI 1 induces a discrete increase of cell death.

## 4. Discussion 

The present study analyzes the effect of very low dose BCG infection on macrophages in terms of cell viability, cell activation, and mitochondrial integrity. Our results demonstrate that although BCG infection at MOI 1 triggers some cell death, there was a clear activation of the viable macrophage. In contrast, BCG infection at MOI 0.1 was sufficient for promoting macrophage activation with a distinct cytokine pattern in the absence of cell death and mitochondrial damage.

Monocytes and macrophages are among the most important cells of the innate immunity involved in host protection against mycobacterial infections. Recent studies have shown that BCG infection induces epigenetic reprogramming in monocytes and macrophages that defines the molecular mechanisms involved in trained immunity conferring a nonspecific protection against a secondary infection [41]. BCG-induced epigenetic modifications in innate cells, such as NOD receptor activation and histone methylation, provide long term functional state of circulating monocytes which may explain the nonspecific beneficial effects already described many years ago for children vaccinated with BCG [42]. In this context, the present work illustrates several of the BCG-induced functional changes on macrophages showing the importance of the selected dosage of BCG which could be relevant in this process.

Studies on human alveolar macrophages have shown that infection with nonpathogenic or pathogenic strains of* M. tuberculosis* (MOI: 5–10), as well as with* M. bovis* BCG, induced apoptosis playing an important role in host-pathogen interaction and contributing to host defense mechanisms against mycobacterial infection [43, 44]. Mycobacteria-induced apoptosis was shown to affect both the infected and uninfected macrophages and be mediated by cell contact and independent of TNF, TGF-*β*, and TLRs [45]. Apoptosis of macrophages during mycobacterial infection has been attributed to different factors including mycobacterial virulence, bacillary load, time points of observation, and the amount of activated cytokines.

Our work shows that the amount of BCG correlates with macrophage apoptosis. Using a BCG-GFP strain, cell death was observed on infected and uninfected macrophages (data not shown) as reported under infection with* M. tuberculosis* [45]. The balance of apoptosis/necrosis is an important mechanism to control intracellular mycobacterial growth and cell activation [46] Nonpathogenic strains such as BCG were reported to induce apoptosis whereas pathogens strain such as H37Rv promoted necrosis in cultured macrophages [44, 47, 48]. However, it has been reported that the balance of apoptosis and necrosis in* M. tuberculosis* infected macrophages depends on bacterial virulence and bacterial load [19].

Macrophage activation involves the induction of cytokines, chemokines, and bactericidal mechanisms. TNF is one of the main cytokines activated during BCG infection in macrophages which has been associated with apoptosis induced by* M. tuberculosis* infection [43]. Mechanisms of evasion developed by virulent* M. tuberculosis* versus avirulent strains involve both TNF production and TNF inactivation by released soluble TNFR2 from activated macrophages [23, 49]. However, human monocytes and macrophages treated with clinically used TNF inhibitors showed that* M. tuberculosis*-induced cell death was independent of TNF and not modulated by TNF inhibition [50]. In mice, macrophage apoptosis has been related to TNF production in the lung of BCG-infected mice using low dose of attenuated versus virulent* M. bovis* which induced higher TNF and higher macrophage apoptosis at early time points but was reversed at late time points of infection indicating a dynamic response* in vivo *[47]. Our data on macrophages show that early apoptosis was independent of intracellular and soluble TNF but correlated with transmembrane TNF expression. Results also show that whereas soluble TNFR1 was independent of BCG dose, soluble TNFR2 was released in a BCG dose dependent manner.

In the attempt to evaluate the main differences in activation between MOI 1 and MOI 0.1, proinflammatory cytokines, molecules other than TNF, have been analyzed. Our data showed that IL-6 was highly produced by BCG infection at MOI 1 while no expression was observed with infection at MOI 0.1. Our data revealed that IL-1*β* and caspase-1 were also differentially modulated by different infectious dosages. BCG infection at MOI 0.1 was more efficient to induce the early production of IL-1*β*, while infection at MOI 1 induced a delay but high level of IL-1*β*. In this context, the microenvironment generated by BCG infection at the two MOIs affected differently macrophage proliferation. Previous data reported that BCG (MOI 3 and MOI 10) failed to stimulate release of IL-1*β* from human macrophages [51]. The different results could be also attributed to different cellular origins. It has been reported that BCG infection of human macrophages induced very low levels of IL-1*β*; however, human monocytes infected with* M. tuberculosis* or BCG delivered comparable levels indicating that the IL-1*β* response is influenced by the host cell type [52]. Similar to IL-1*β*, we observed that MOI 0.1 was a better stimulus to induce MCP-1 and KC expression at early time after infection. However, at late time points BCG infection at MOI 1 induced higher protein amounts than infection at MOI 0.1. From these data we conclude that MOI 0.1 is a good early inducer of two important chemokines MCP-1 and KC which are relevant in maintaining the integrity of granuloma in asymptomatic individuals and also mediate host defense via activation of transcription factors, MAPK and adhesion molecules [53, 54]. We have then analyzed transcription factors and found that NF*κ*B and ASK1 were similarly activated at early time by both BCG MOIs and at 18 h, and using MOI 0.1, cytokines production and NF*κ*B were downregulated. No differences were found in ERK1/2 phosphorylation patterns at the two infection dosages. In addition, both MOIs were able to activate bactericidal mechanisms required for bacterial elimination and as expected, MOI 1 induced higher iNOS protein and NO production.

Previous report has shown that mycobacterial infection in macrophages caused mitochondrial perturbation. Comparison of the effects of virulent versus avirulent* M. tuberculosis* strains showed that virulent strain increased mitochondrial activity whereas avirulent strains resulted in mitochondria exhaustion suggesting that virulent strains could maintain a niche for sustained survival [40]. Our data show that both BCG infections transiently increased the amount of the mitochondrial protein TOM40 suggesting a transient increase in mitochondrial mass. This could be a consequence of the stress induced by the infection; however more experiments will be necessary to test this hypothesis.

In this study, we also used parameters such as cellular granularity and size and chromatin condensation that classically has been described as indicators of cellular integrity [55, 56]. Even if macrophages under condition MOI 1 showed a discrete loss of SSC compared to uninfected macrophages, the condensed chromatin and mitochondria were not affected. We concluded that low BCG doses are enough to activate macrophages and to maintain cellular viability and mitochondrial integrity.

Although our study has limitations due to the fact that it has been developed in a cell line, it can be considered that data provide new insights into macrophage activation by BCG infection with very low number of bacilli that can be used as an* in vitro* infection system and applied to studies on macrophage early activation mechanism maintaining cell viability. More extensive studies are needed to confirm that very low BCG dose may induce changes in innate cells and provide beneficial effects. In support to our data, the use of one-half or one-third of BCG for standard instillation has recently been proposed. A study in NMIBC-patients treated with low dose BCG had lower toxicity and higher quality of life compared with NMIBC-patients instilled with standard-dose [57, 58]. Thus, low dose BCG can be applied to different BCG research fields of interest including mycobacterial infections, cancer immunotherapy, and prevention of autoimmunity and allergies [59].

## Supplementary Material

Supplementary Figure 1: Pro-caspase 1 is detected earlier in macrophages infected at MOI 0.1 than MOI 1. Western blot analyses for pro-caspase-1 p45, caspase-1 p20 and tubulin of cells infected at MOI 1 and MOI 0.1 at different time points. Bars indicate mean +/- SEM from three independent experiments. ∗∗P<0.01, ∗P<0.03.
