# Supplementary material for: Low Dose BCG Infection as a Model for Macrophage Activation Maintaining Cell Viability
Source: J Immunol Res. 2016 Oct 19;2016:4048235. doi: 10.1155/2016/4048235 (PMC5090099; doi:10.1155/2016/4048235)
Supplement: Supplementary file 1 — Supplementary Figure 1: Pro-caspase 1 is detected earlier in macrophages infected at MOI 0.1 than MOI 1. Western blot analyses for pro-caspase-1 p45, caspase-1 p20 and tubulin of cells infected at MOI 1 and MOI 0.1 at different time points. Bars indicate mean +/- SEM from three independent experiments. ∗∗P<0.01, ∗P<0.03. [file 4048235.f1.docx]

Pro–Caspase 1

Uninfected

0.1

0.1

0.1

1

1

1

MOI

2h

5h

18h

Tubulin


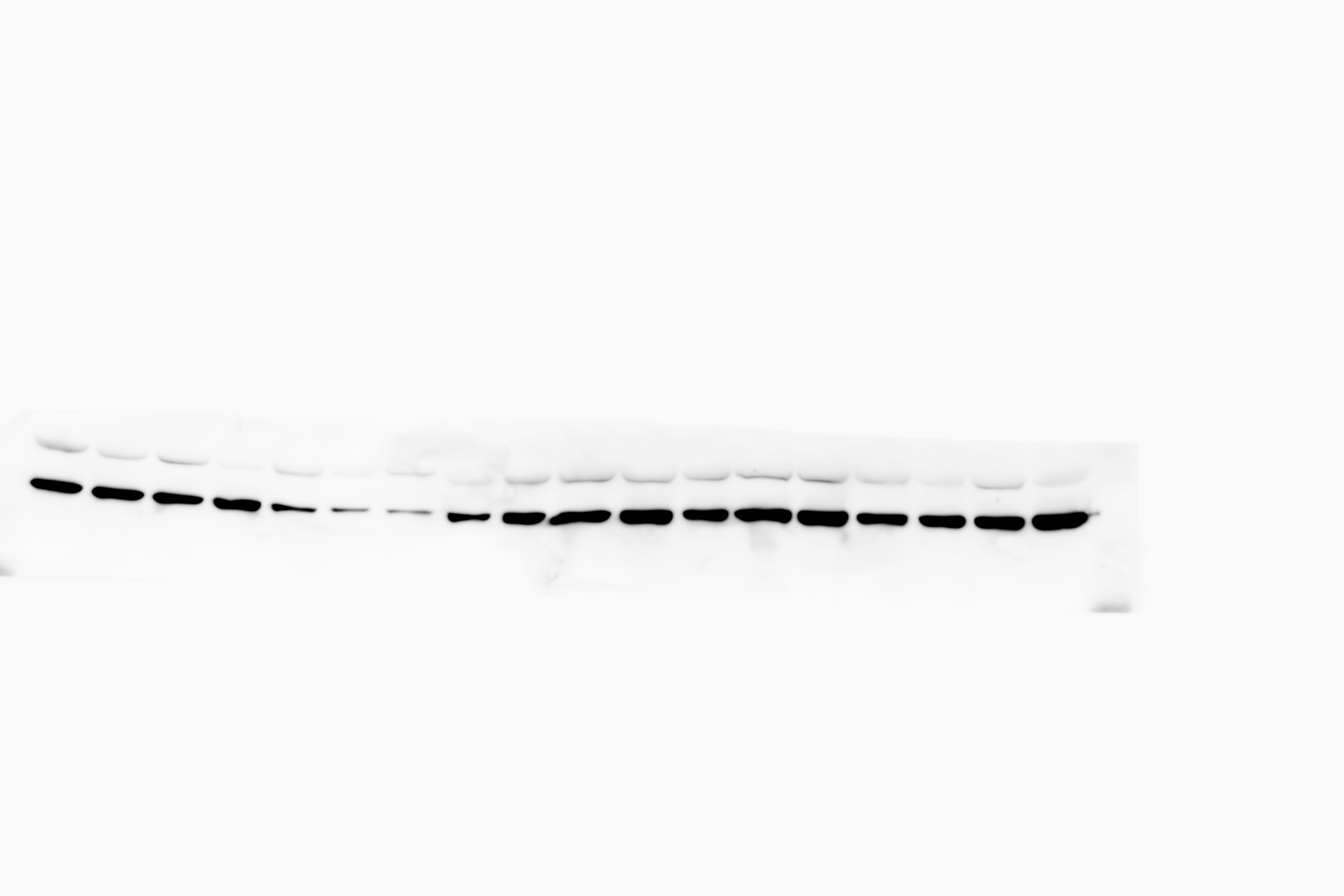

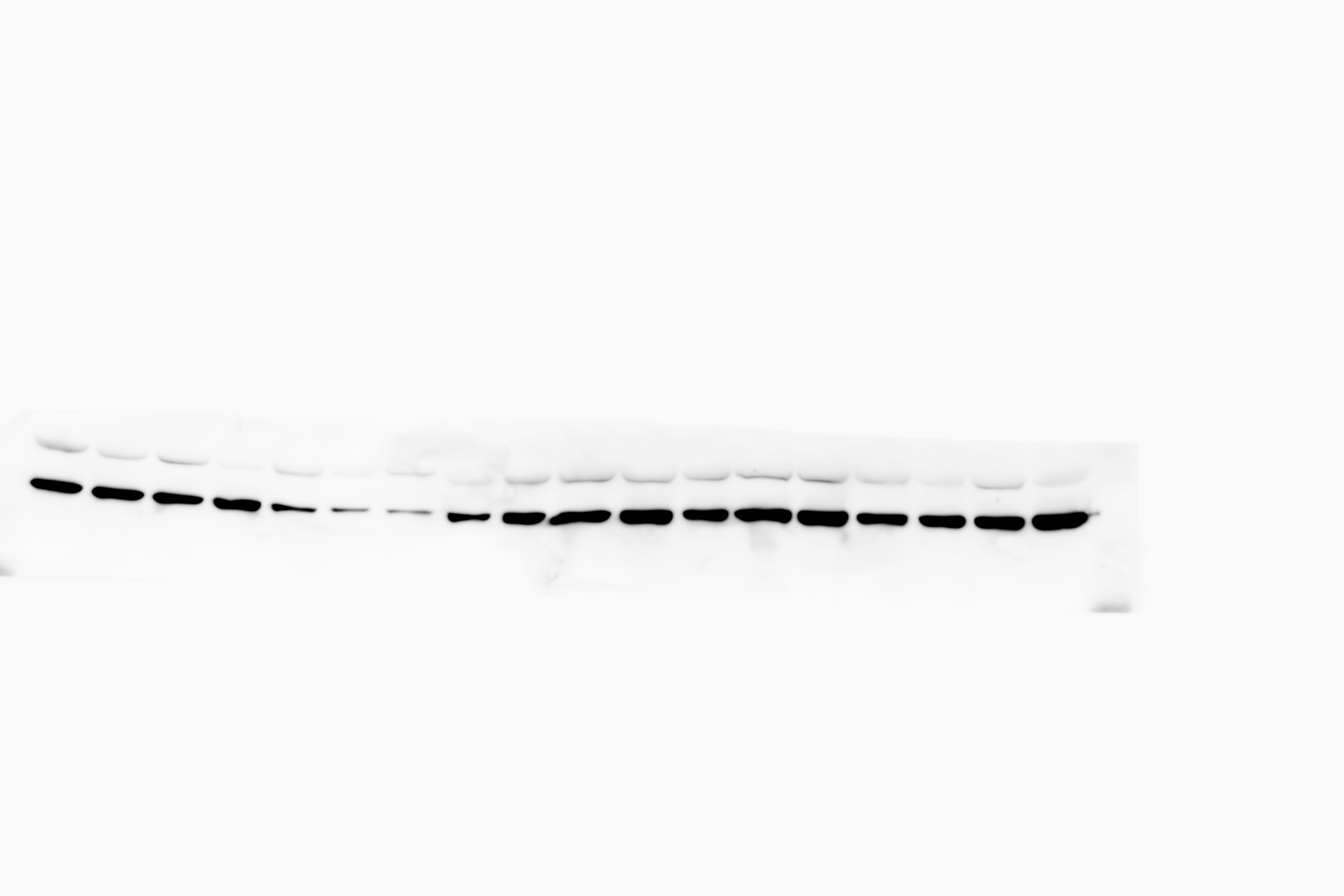

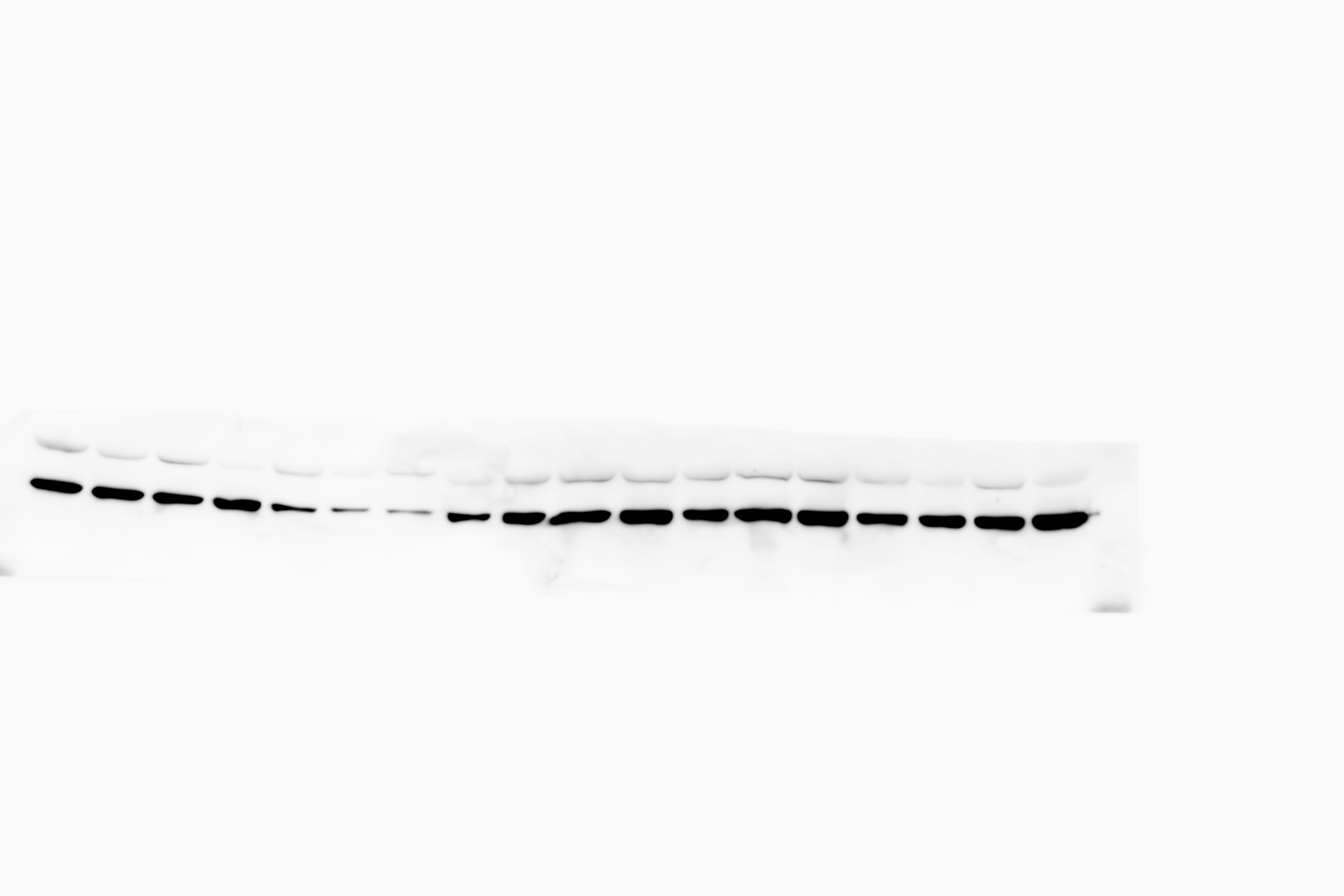

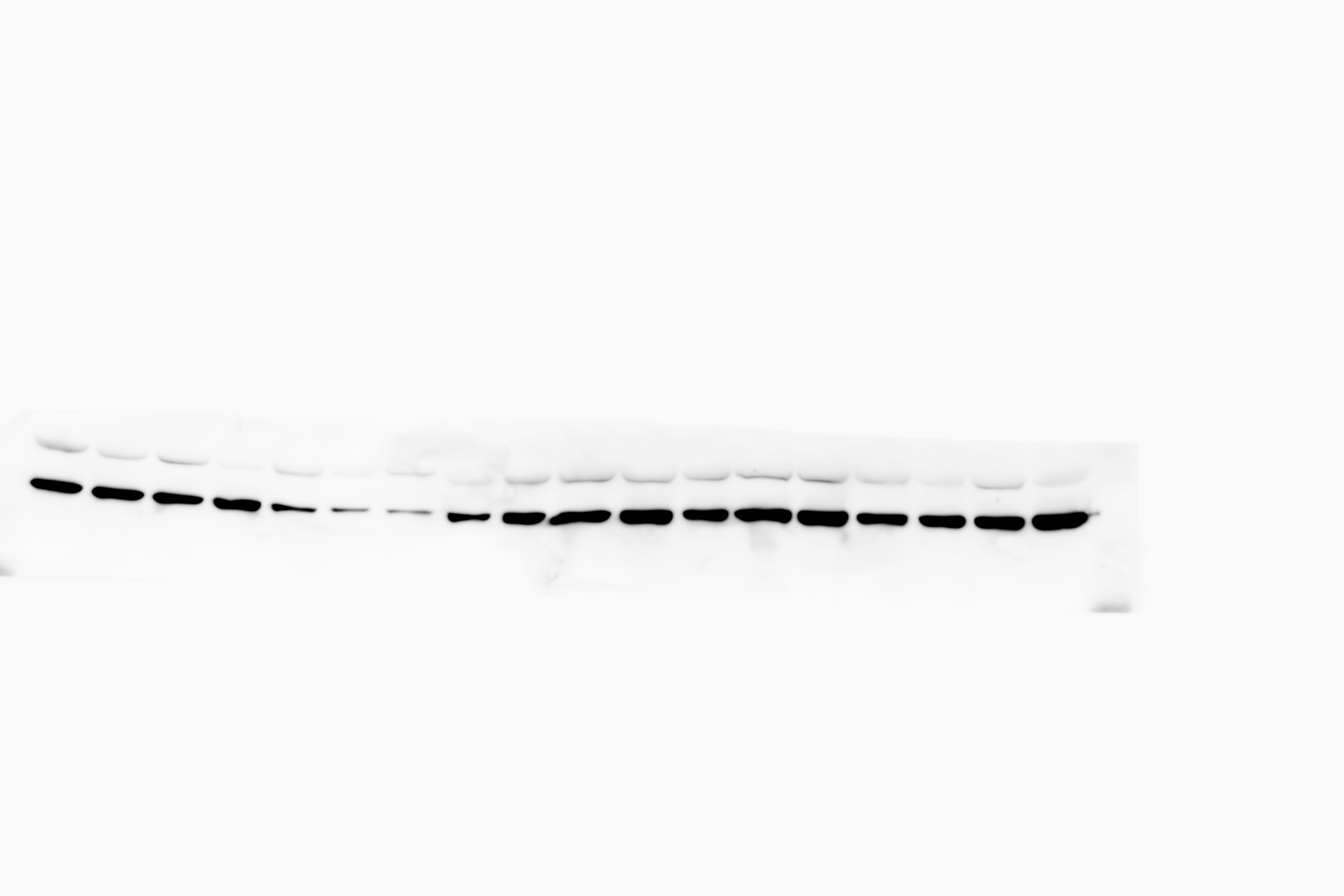

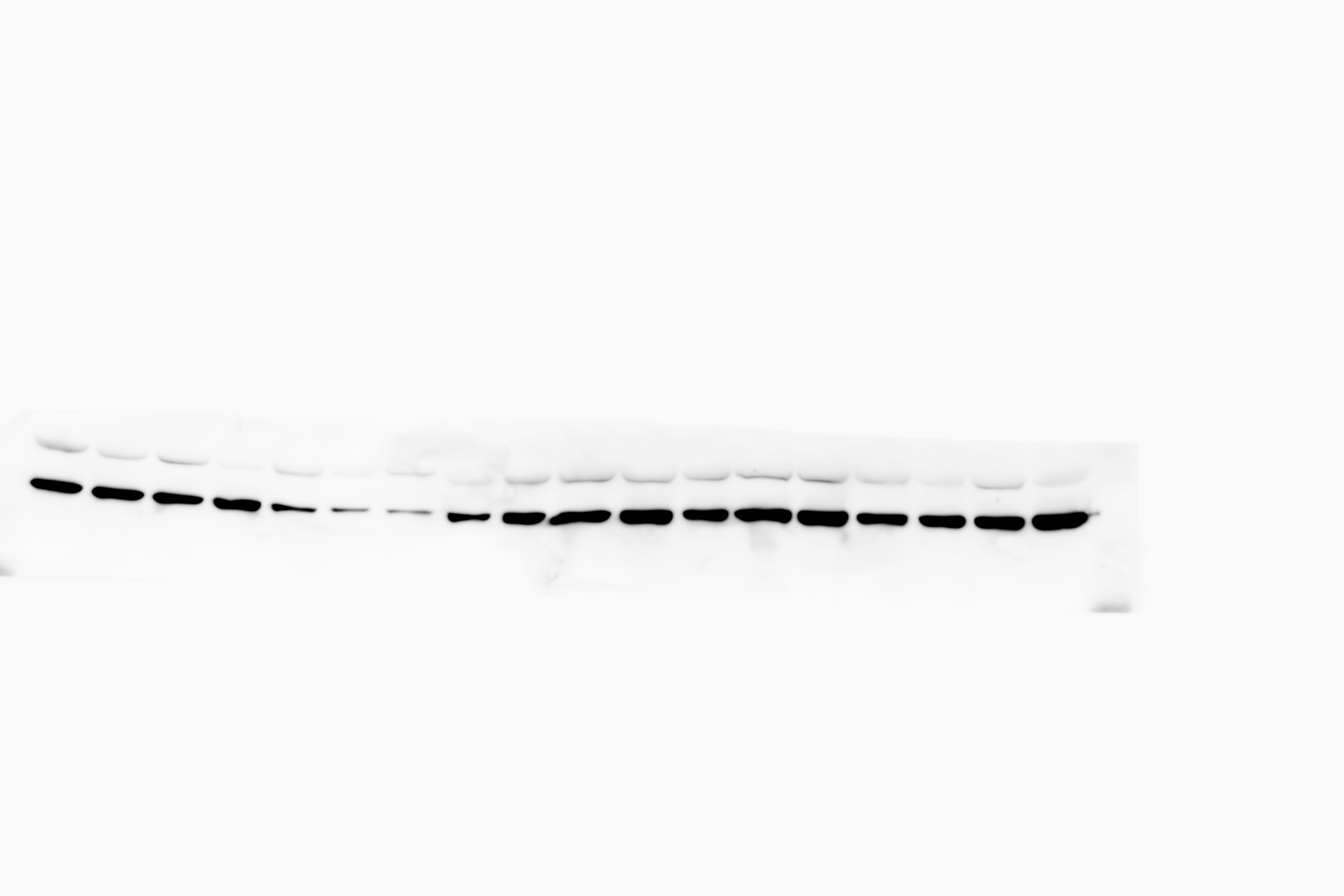

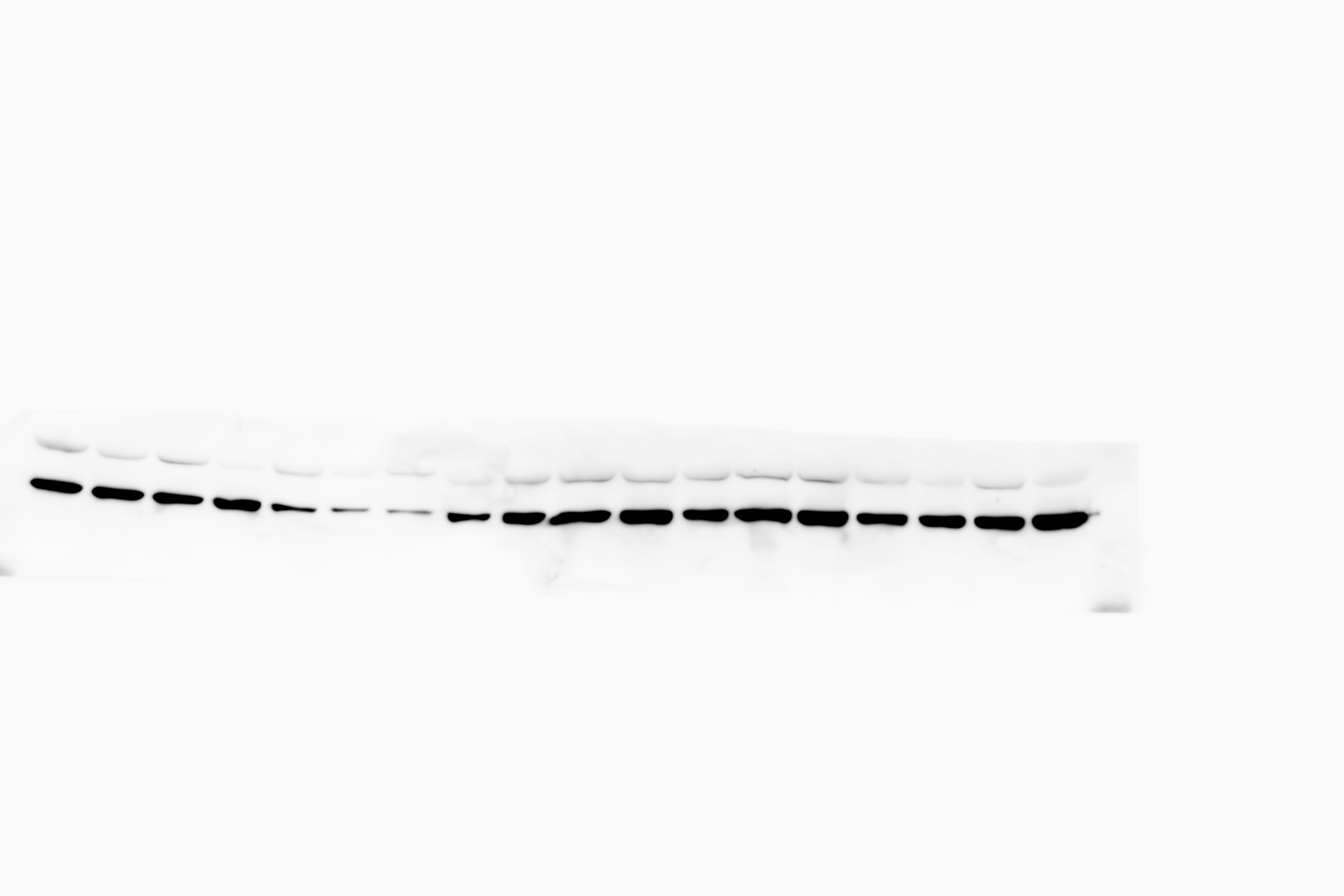

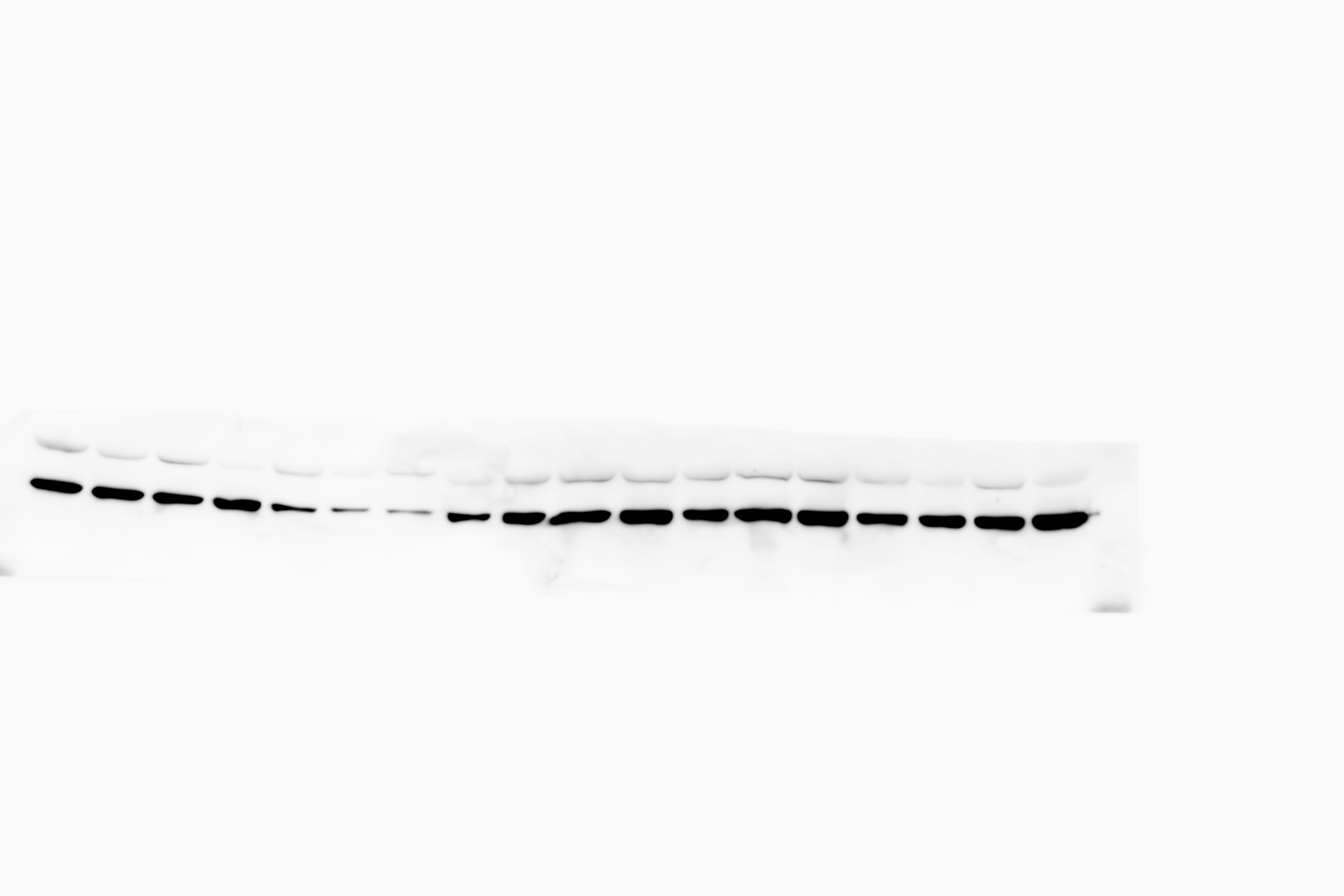

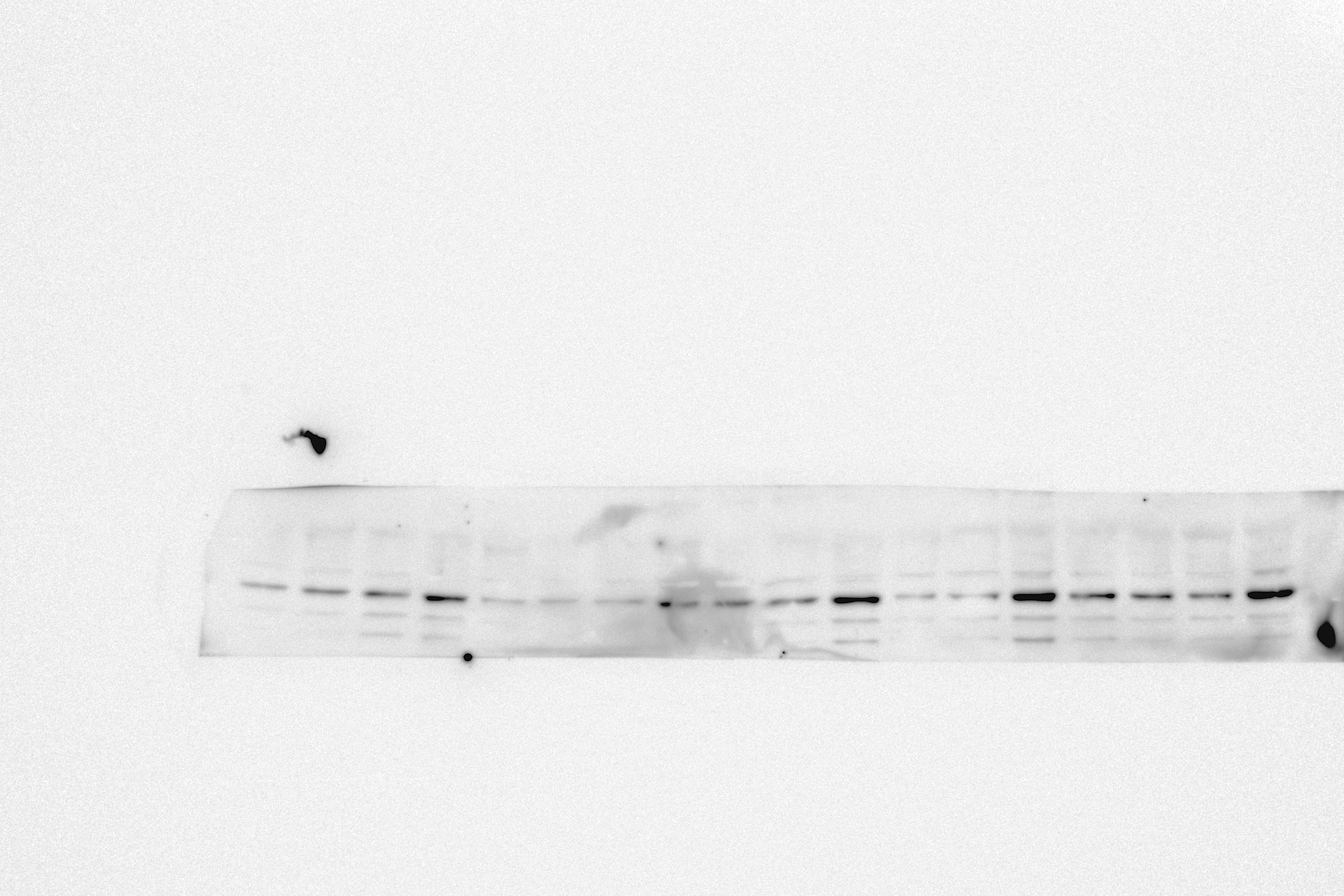

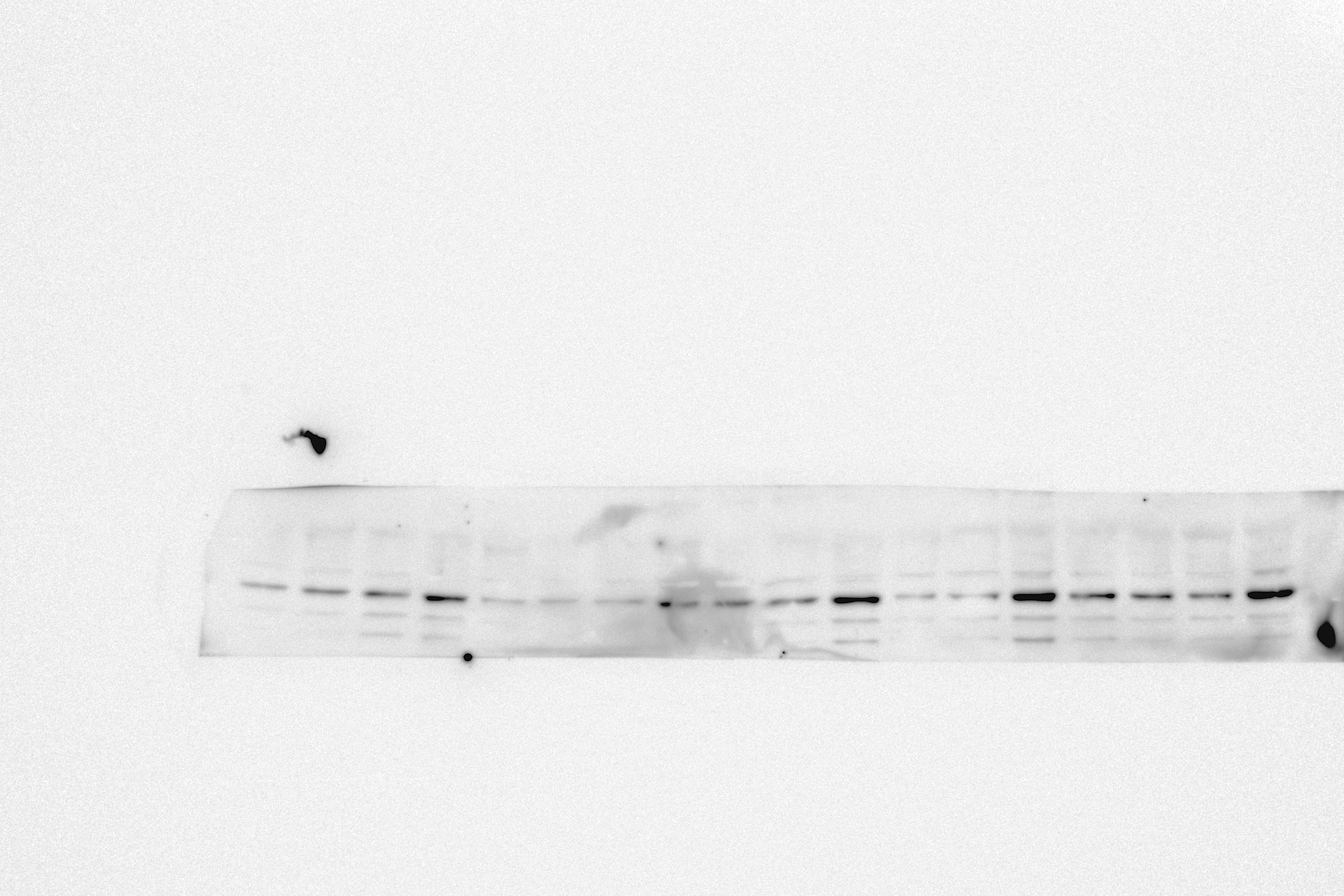

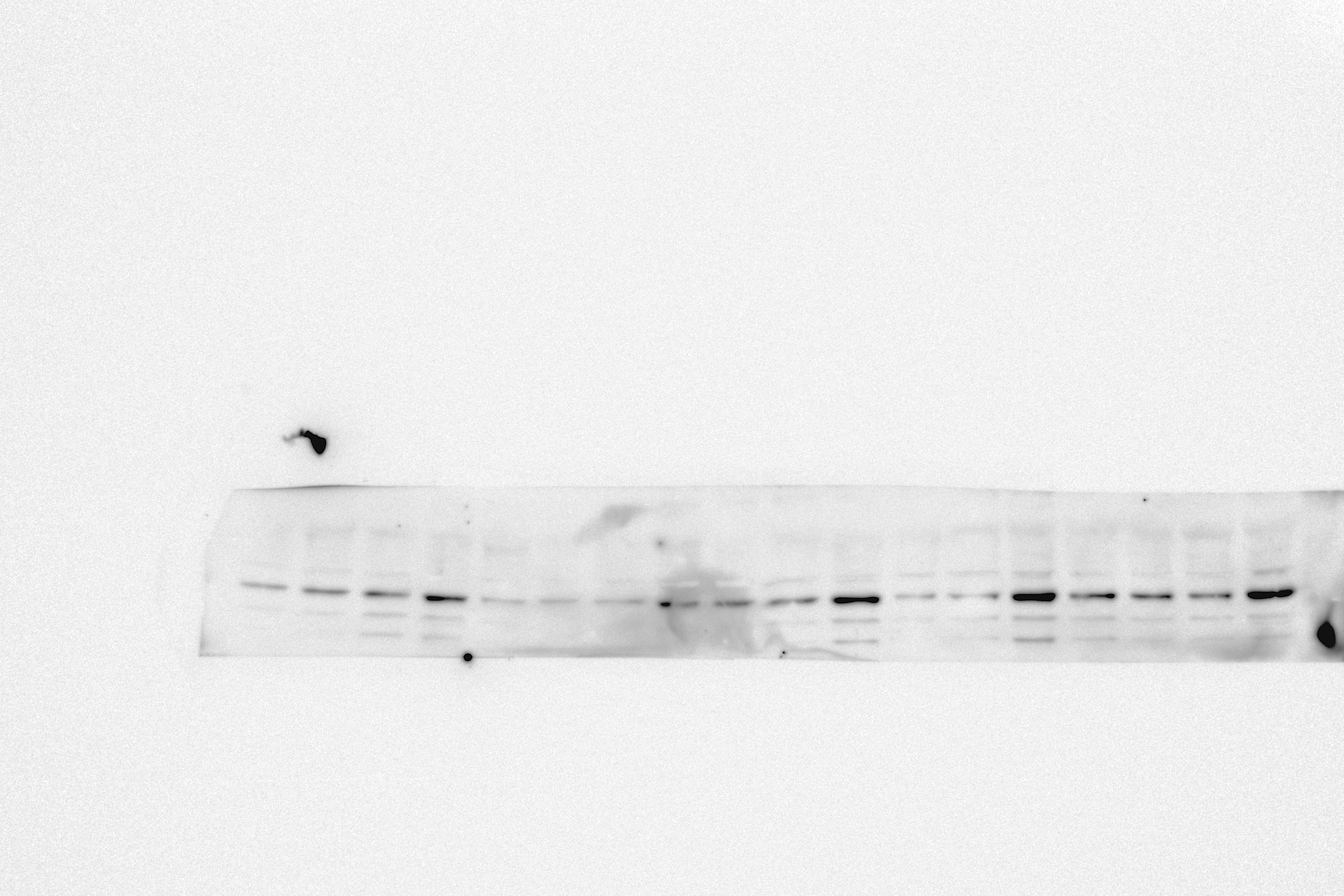

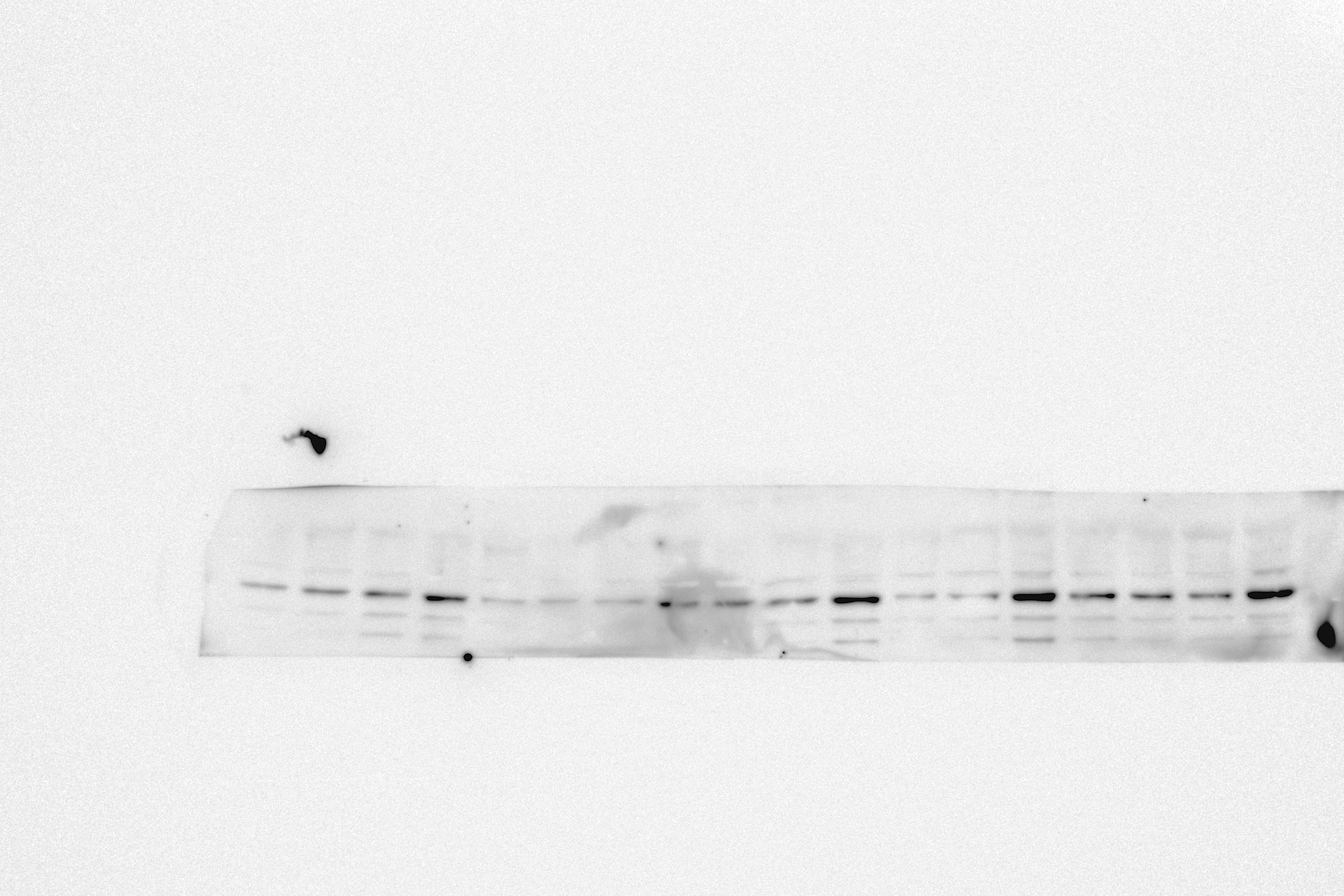

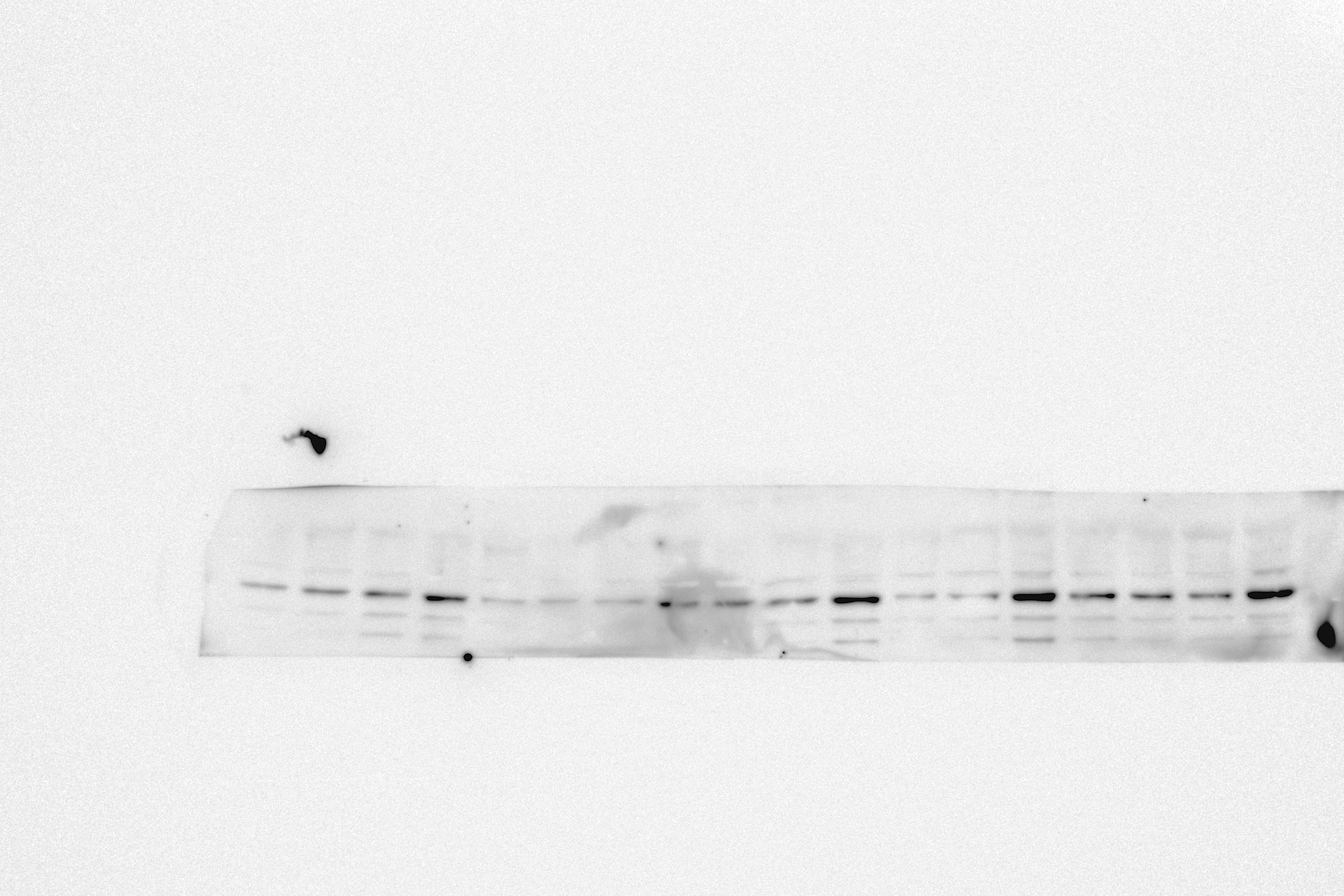

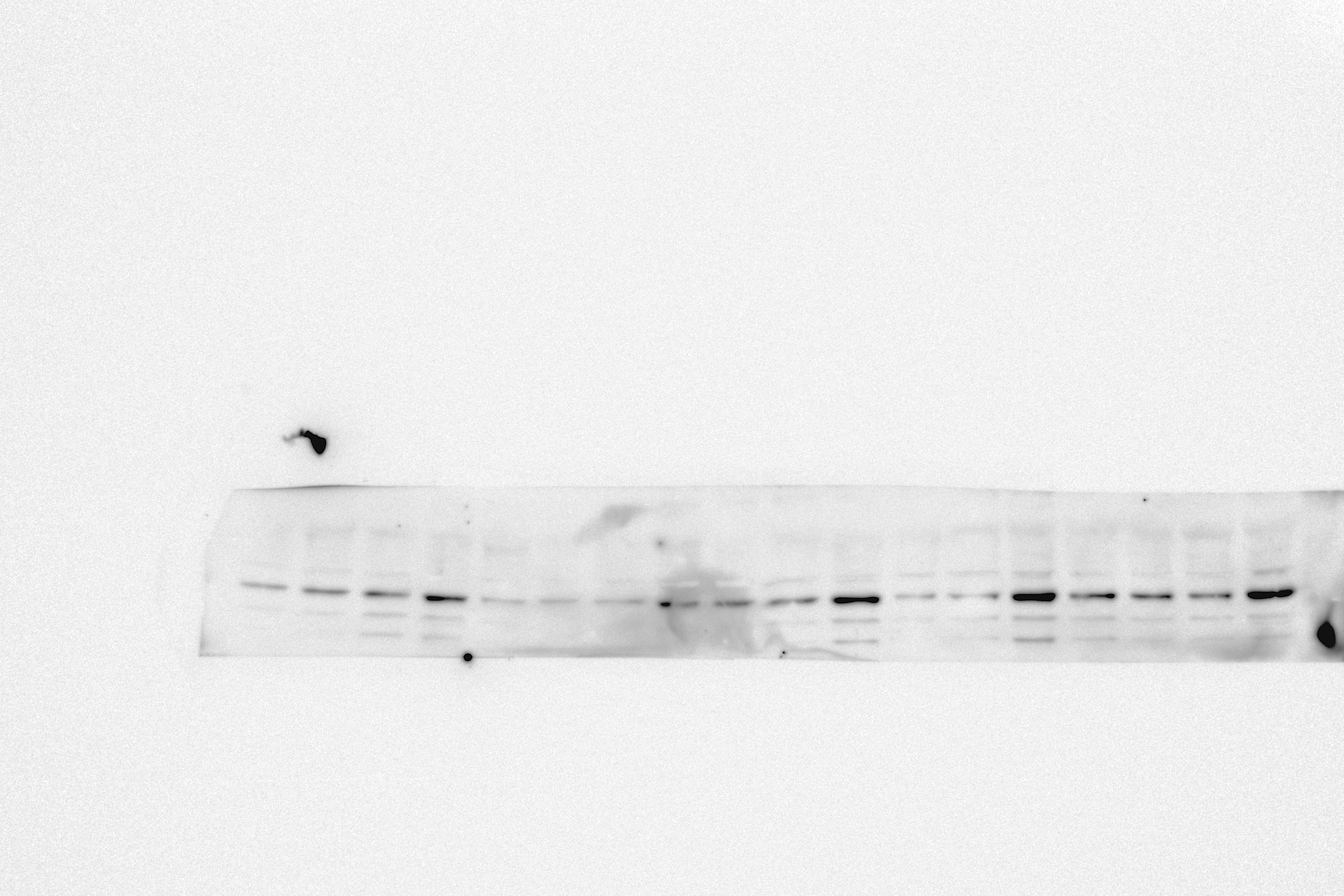

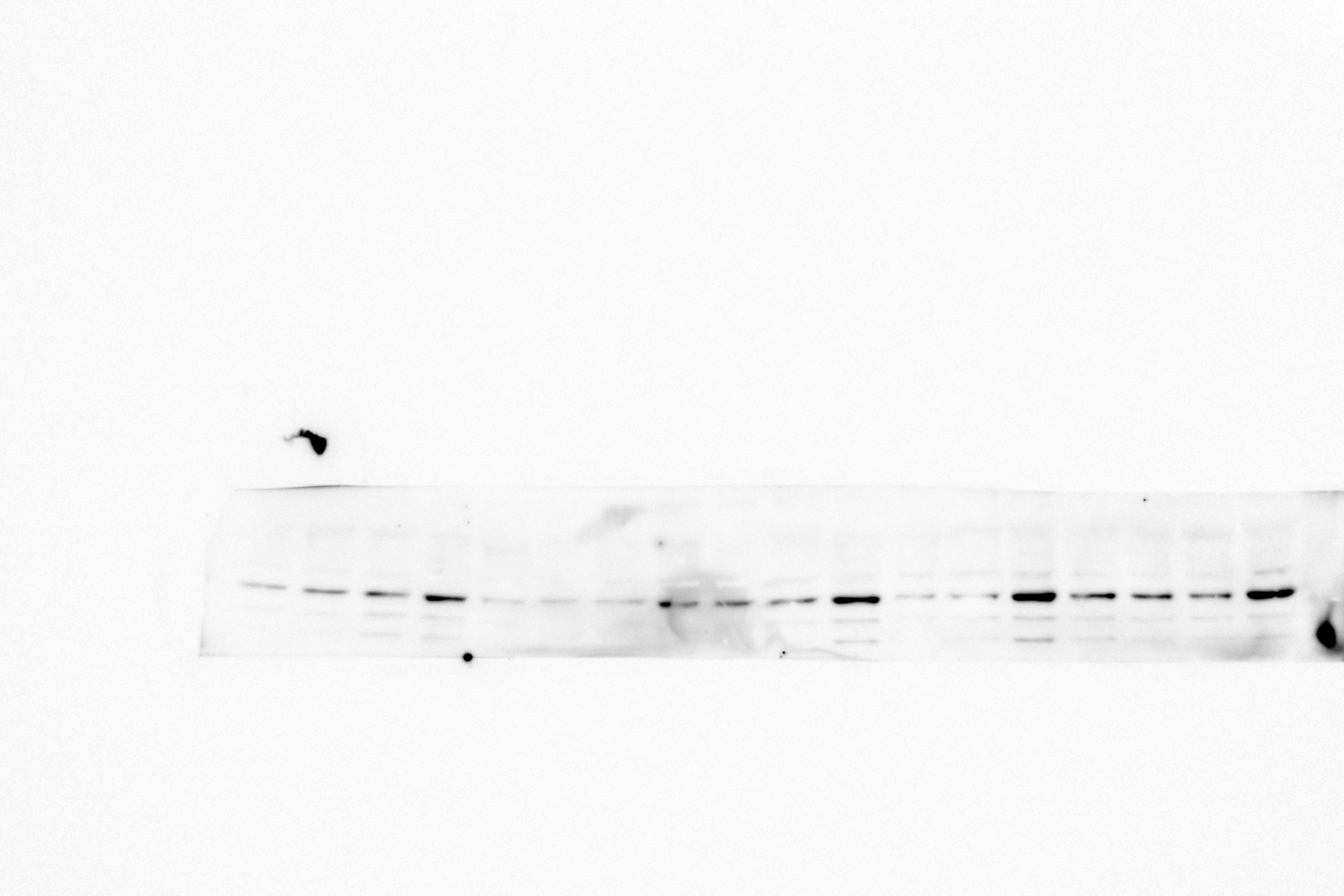

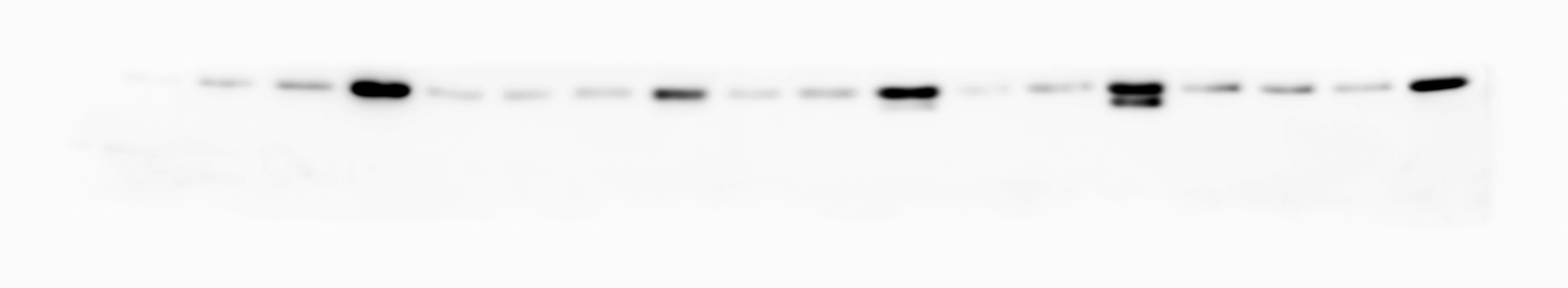

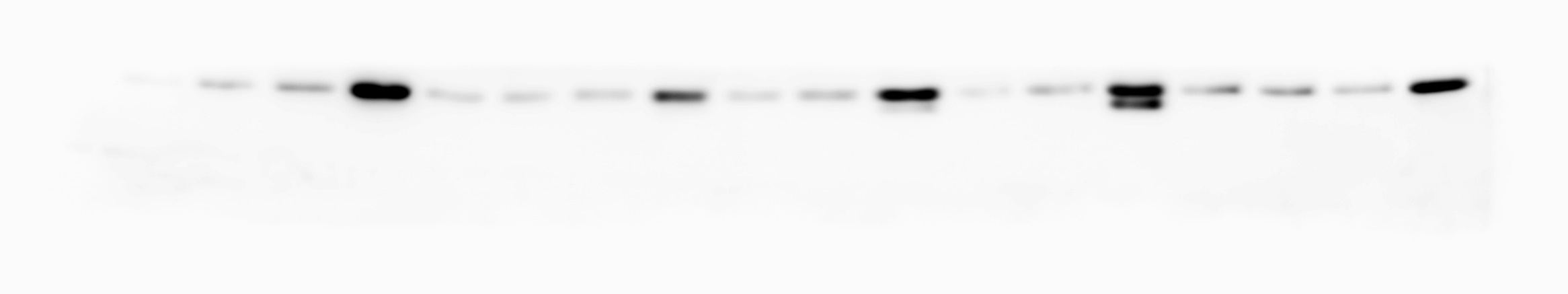

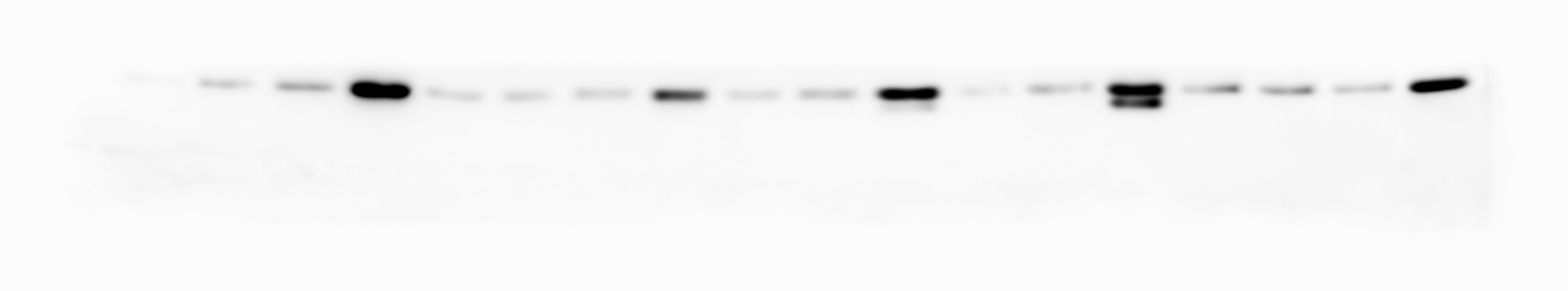

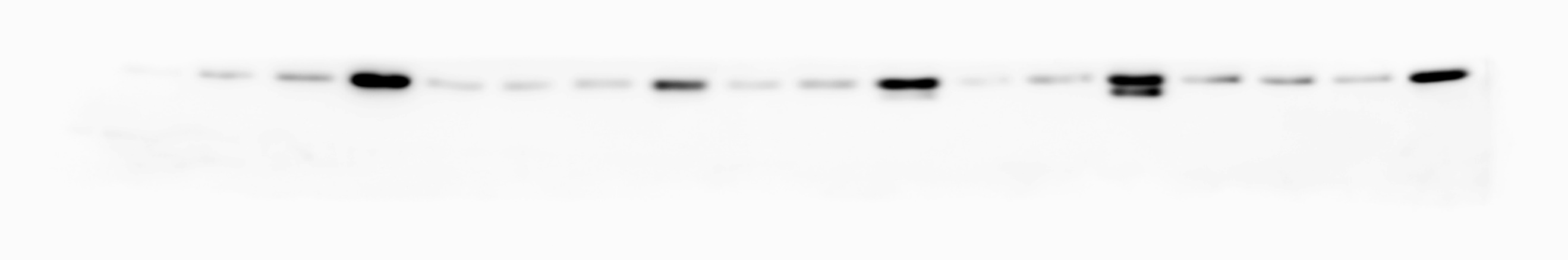

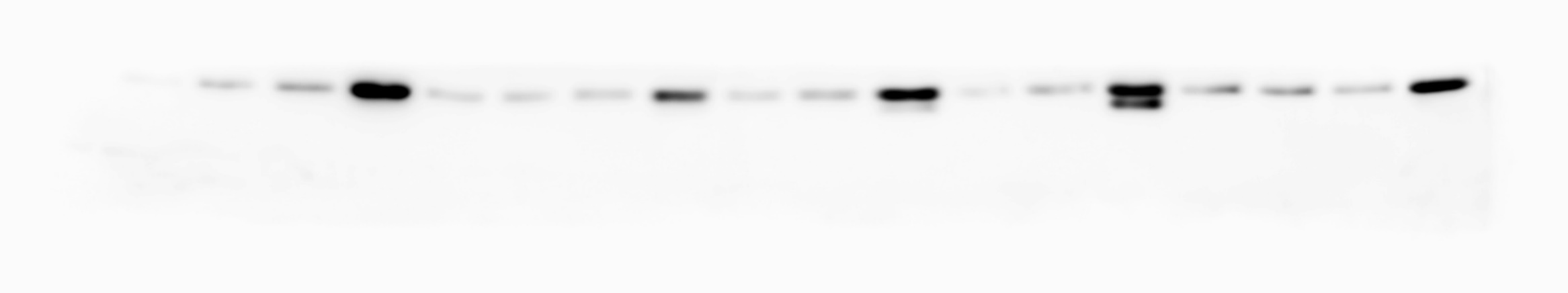

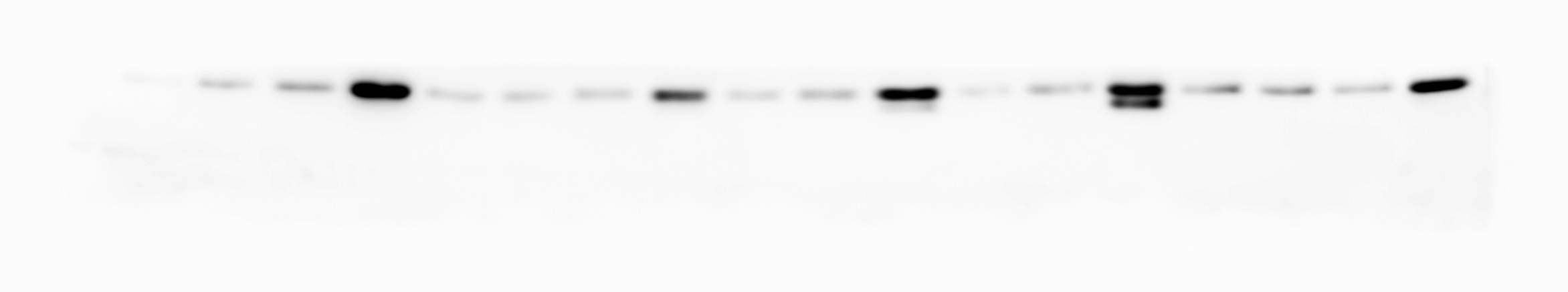

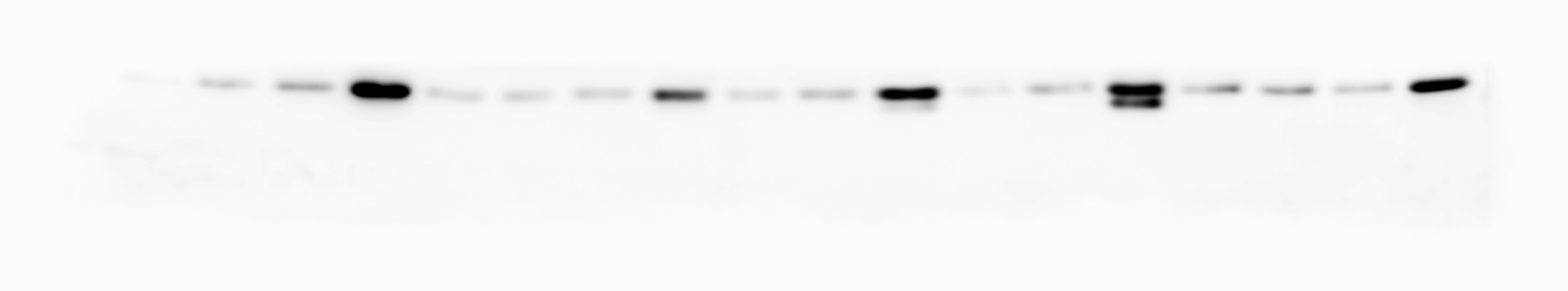


Caspase 1 p20

**Supplementary Figure 1. Pro Caspase-1 is detected earlier in macrophages infected at MOI 0.1 than MOI 1.** Western blot analyses for pro-caspase-1 p45, caspase-1 p20 and tubulin of cells infected at MOI 1 and MOI 0.1 at different time points. Bars indicate mean +/- SEM from three independent experiments. ***P*<0.01, **P*<0.03.
